# Analysis of six consecutive waves of ICU-admitted COVID-19 patients: key findings and insights from a Portuguese population

**DOI:** 10.1007/s11357-024-01410-x

**Published:** 2024-11-14

**Authors:** Cristiana P. Von Rekowski, Iola Pinto, Tiago A. H. Fonseca, Rúben Araújo, Cecília R. C. Calado, Luís Bento

**Affiliations:** 1 NMS – NOVA Medical School, FCM – Faculdade de Ciências Médicas, Universidade NOVA de Lisboa, Campo Mártires da Pátria 130, 1169-056 Lisbon, Portugal; 2 ISEL – Instituto Superior de Engenharia de Lisboa, Instituto Politécnico de Lisboa, Rua Conselheiro Emídio Navarro 1, 1959-007 Lisbon, Portugal; 3 CHRC – Comprehensive Health Research Centre, Universidade NOVA de Lisboa, 1150-082 Lisbon, Portugal; 4 Department of Mathematics, ISEL – Instituto Superior de Engenharia de Lisboa, Instituto Politécnico de Lisboa, Rua Conselheiro Emídio Navarro 1, 1959-007 Lisbon, Portugal; 5 NOVA Math – Center for Mathematics and Applications, NOVA FCT – NOVA School of Science and Technology, Universidade NOVA de Lisboa, Largo da Torre, 2829-516 Caparica, Portugal; 6 iBB – Institute for Bioengineering and Biosciences, i4HB – The Associate Laboratory Institute for Health and Bioeconomy, IST – Instituto Superior Técnico, Universidade de Lisboa, Av. Rovisco Pais, 1049-001 Lisbon, Portugal; 7 Intensive Care Department, ULSSJ – Unidade Local de Saúde São José, Rua José António Serrano, 1150-199 Lisbon, Portugal; 8 Integrated Pathophysiological Mechanisms, CHRC – Comprehensive Health Research Centre, NMS – NOVA Medical School, FCM – Faculdade de Ciências Médicas, Universidade NOVA de Lisboa, Campo Mártires da Pátria 130, 1169-056 Lisbon, Portugal

**Keywords:** COVID-19 waves, ICU, COVID-19 vaccination, Early mortality, Late mortality

## Abstract

Identifying high-risk patients, particularly in intensive care units (ICUs), enhances treatment and reduces severe outcomes. Since the pandemic, numerous studies have examined COVID-19 patient profiles and factors linked to increased mortality. Despite six pandemic waves, to the best of our knowledge, there is no extensive comparative analysis of patients’ characteristics across these waves in Portugal. Thus, we aimed to analyze the demographic and clinical features of 1041 COVID-19 patients admitted to an ICU and their relationship with the different SARS-Cov-2 variants in Portugal. Additionally, we conducted an in-depth examination of factors contributing to early and late mortality by analyzing clinical data and laboratory results from the first 72 h of ICU admission. Our findings revealed a notable decline in ICU admissions due to COVID-19, with the highest mortality rates observed during the second and third waves. Furthermore, immunization could have significantly contributed to the reduction in the median age of ICU-admitted patients and the severity of their conditions. The factors contributing to early and late mortality differed. Age, wave number, D-dimers, and procalcitonin were independently associated with the risk of early death. As a measure of discriminative power for the derived multivariable model, an AUC of 0.825 (*p* < 0.001; 95% CI, 0.719–0.931) was obtained. For late mortality, a model incorporating age, wave number, hematologic cancer, C-reactive protein, lactate dehydrogenase, and platelet counts resulted in an AUC of 0.795 (*p* < 0.001; 95% CI, 0.759–0.831). These findings underscore the importance of conducting comprehensive analyses across pandemic waves to better understand the dynamics of COVID-19.

**Supplementary Information:**

The online version contains supplementary material available at 10.1007/s11357-024-01410-x.

## Background

The first cases of Coronavirus Disease 2019 (COVID-19), caused by severe acute respiratory syndrome coronavirus 2 (SARS-CoV-2), were documented in Wuhan, China. Following that, the disease spread rapidly to a number of countries, giving rise to several variants that posed significant threats to global health (Table 1), ultimately leading to the declaration of a pandemic on March 11, 2020 [1]. During the early months of the pandemic, a variant carrying the Spike D614G mutation became dominant, being linked with increased transmissibility [2]. With the increasing number of documented cases, Portugal implemented the first measures to contain virus transmission by declaring a state of emergency. These included measures such as prohibiting intermunicipal travel and closing airports [3]. Throughout this first wave of the pandemic, the majority of identified viruses belonged to clades 20A (40%) and 20B (46%) [4]. By the end of March 2020, a sub-clade carrying the D839Y mutation was identified in northern Portugal, causing one of the largest transmission chains in the country. This mutation, first documented in Italy (Lombardy), is rarely found in other countries, suggesting that its emergence in Portugal may have originated there [2]. The onset of the second wave began in August 2020, when many of the pandemic containment measures were eased due to the evident decrease in COVID-19 cases. At this time, all identified viruses were categorized under clade 20B [4]. Nevertheless, European travel resumed over the summer months, and the absence of screening and containment measures led to the emergence and rapid spread of a new variant across the continent, known as 20E (EU1). By October, 20E was identified in 70% of the screened samples in Portugal, and these elevated percentages persisted almost until the end of the second wave [4, 5]. As cases continued to increase, between October and November 2020, containment measures for the virus were once again intensified, prompting the declaration of a new state of emergency and implementation of lockdown measures in municipal districts [3]. Following the WHO Emergency Use Listing of the Pfizer/BioNtech Comirnaty vaccine, Portugal initiated its COVID-19 vaccination program in late December 2020, coinciding with the end of the second wave [6]. However, the program faced constraints due to limited vaccine availability [3]. Additional vaccines were introduced in January 2021, namely Moderna (mRNA-1273), followed by two AstraZeneca/Oxford vaccines in February 2021, and the Janssen/Ad26.COV2.S vaccine in March 2021[6].

**Table 1:** Key characteristics of major COVID-19 variants in Portugal and associated focal public health measures

Wave	Start (month D, Yr.)^a^	End (month D, Yr.)^a^	Variants^b^ [13]	Variant descendance [13]	Emergence/initial concentration [13]	Focal public health measures [13, 15–17]
First	March 10, 2020	August 22, 2020	20A, B.1 20B, B.1.1	20A derived from 19A, B (Wuhan, China) 20B derived from 20A, B.1	Global (Early 2020)	State of Emergency declared (March 18, 2020) Intermunicipal travel ban and airport closures (April 2, 2020) End of State of Emergency and reopening of some establishments (May 2020) Lifting of nearly all containment measures (until the end of August 2020)
Second	August 23, 2020	December 19, 2020	20E (EU1), B.1.177	Derived from 20A, B.1	Spain (Summer 2020)/Europe	Further easing of measures (September 2020) State of Alert declared (September 15, 2020) Municipal lockdown measures declared (October 31, 2020) New State of Emergency (November 9, 2020)
Third	December 20, 2020	May 31, 2021	20I (Alpha, V1), B.1.1.7	Derived from 20B, B.1.1	UK (September 2020)/Europe	Travel restrictions (December 20, 2020) Vaccination program start (late December 2020) National lockdown (January 15, 2021) National Testing Strategy update and acceleration of the vaccination plan implementation (February 26, 2021) Gradual reopening of nurseries, schools, sports, and restaurants (March to May 2021) End of Emergency State (April 30, 2021) Declaration of Calamity State (until May 30, 2021)
Fourth	June 1, 2021	October 31, 2021	21J (Delta)	Derived from 21A (Delta), B.1.617.2	India (October 2020)/Global	Acceleration of the vaccination plan implementation Varying restrictions across municipalities based on infection rates (June 17, 2021) Phased Reopening Linked to Vaccination Milestones (August 2021), aiming for full reopening by mid-September
Fifth	November 1, 2021	March 30, 2022	21K (Omicron), BA.1 21L (Omicron), BA.2	Derived from 21M (Omicron), B.1.1.529	South Africa (November 2021)/Western Europe	Free testing increase Holiday restrictions: mandatory remote work, closures, and negative test requirements (December 23, 2021)
Sixth	April 1, 2022	August 8, 2022	22B (Omicron), BA.5 21L (Omicron), BA.2	Derived from 21M (Omicron), B.1.1.529	South Africa (Early 2022)/Europe	End of restrictions, including the removal of quarantine for risk contacts, remote work recommendations, capacity limits, digital certificate requirements, and negative tests for large events, while maintaining a Nationwide Situation of Alert (February 2, 2022)

^a^The start and end dates of the waves are according to what was established by the ICUs of *Unidade Local de Saúde São José*, Lisbon (see the “Methods” section)

^b^Nextstain clade, followed by Pango lineage

The third wave was marked by the emergence of the 20I (Alpha V1) variant, first documented in the UK in September 2020. It rapidly displaced the 20E variant in early 2021, peaking at 87% of screened cases by April. Additionally, other VOCs like the 20J (Gamma V3) (P.1 lineage) and 20H (Beta V2) (B.1.351 lineage) were identified, primarily in April (4.3%) and March (2.5%) 2021, respectively [7]. Around this time, Portugal’s vaccine task force intensified efforts to boost immunization rates by expanding supply, broadening target groups, and establishing numerous vaccination centers, following a prioritized hierarchy based on exposure risk, age, high-risk health conditions, and essential roles. Concurrently, containment measures were once again eased, leading to the reopening of public spaces such as schools, some services, and later restaurants [3]. In June 2021, the 21J (Delta) variant started to dominate, representing over 50% of all detected cases, shaping the onset of the fourth wave. From August until the conclusion of the fourth wave, it was detected in 94–97% of national samples, demonstrating its extensive coverage [7]. Interestingly, by the end of August 2021, 74% of the Portuguese population had received two doses, or the equivalent, of the COVID-19 vaccine, and 84% had received their first dose, surpassing the European Union’s average [3].

On November 24, 2021, yet another variant was identified in South Africa, namely Omicron (B.1.1.529 lineage), quickly outpacing the highly contagious Delta variant [8]. Compared to others, the omicron’s transmissibility and vaccination resistance were increased as a result of its high number of mutations [9]. Meanwhile, in Portugal, alongside numerous reported cases of the Delta variant until the end of the year, the detection of the Omicron 21K variant in November 2021 marked the onset of the fifth COVID-19 wave. By the end of January 2022, 21K had been identified in 82% of all screened samples [10]. Shortly after, cases of the 21L variant began to increase, accounting for most cases in Portugal by the end of February and reaching its highest detection rate in March 2022, when it was identified in 82% of all samples [10]. In early April, the 22B variant was detected for the first time, characterizing Portugal’s sixth COVID-19 wave. By May, 22B had become the most prevalent variant, accounting for approximately 56% of identified cases in certain weeks, while 43% were still linked to the 21L variant. After June 2022, the frequency of 22B rose significantly, representing 73–97% of all detected cases until the end of the wave [11]. Since the end of the pandemic, the 24C variant (Omicron, KP.3) considerably increased in circulation all around the world, particularly in Europe and America, and was detected in 72% of the analyzed samples in Portugal as of July 2024 [12, 13]. This surge led to an increase in COVID-19 cases in Portugal, with an average of 400 new cases and 12 deaths per day, figures that tripled since May 2024 [14].

Given the persistent global prevalence of COVID-19, along with the recurrent emergence of new variants, several studies aimed to provide high-quality data on COVID-19 patient profiles across various populations. The examination of comorbidities, the impact of blood biomarkers, and other clinical characteristics on adverse outcomes, including mortality, has helped identify high-risk patients and improve their diagnosis and treatment [18–20]. However, most of these studies rely on a broader view of the pandemic instead of providing a better understanding of each wave of infection. Analyzing clinical characteristics of patients, while considering the effects of immunization and other public health measures, is essential for gaining insights into each pandemic wave. This focus was evident early in the pandemic, when some studies compared the initial COVID-19 variants, such as alpha and delta [21–25]. However, research comparing more than three pandemic waves remains limited. As previously described, Portugal experienced six waves of the pandemic; however, there appears to be no comparative analysis of COVID-19 patient characteristics between these waves. Furthermore, examining patient outcomes during these waves, particularly mortality and its timing, would be essential for a clearer understanding of the impact of each variant, along with the factors that contributed to those outcomes. To the best of our knowledge, current predictions of COVID-19 mortality are mostly based on data from overall SARS-CoV-2 infections; thus, COVID-19 waves are rarely acknowledged [26]. Moreover, there appears to be no analysis related to the timing of death or to the factors that may have contributed to an earlier or later outcome. Thus, the primary goal of this study was to describe and compare the characteristics and outcomes of a Portuguese population of COVID-19 patients admitted to an intensive care unit (ICU), across six waves of the pandemic. Additionally, the risk factors associated with both early and late mortality were analyzed, using data collected within the initial 72 h of ICU admission.

## Methods

We conducted a retrospective observational study on COVID-19 patients admitted to the ICUs of *Unidade Local de Saúde São José* (ULSSJ), Lisbon, during the six waves of the pandemic. Patient data was collected in compliance with patient anonymity and legal and ethical requirements, including written informed consent and approval by the Ethics Committee of ULSSJ (1043/2021, 20 May 2020), in the scope of the Predictive Models of COVID-19 Outcomes for Higher Risk Patients Towards a Precision Medicine (PREMO) project. Demographic and clinical data, including daily blood analyses performed in the ICU, was obtained from the hospitals’ electronic medical record system between March 2020 and August 2022 for all COVID-19 patients. The diagnosis was confirmed with real-time polymerase chain reaction tests (RT-PCR) for SARS-CoV-2. After collecting all data, patients under 18 years of age and those who were still hospitalized at the time the data was collected were excluded. Patients lacking records of routine blood analyses performed in the ICU were also excluded from the study. All remaining COVID-19 patients with dates of RT-PCR tests for SARS-CoV-2, ICU admission and discharge, and hospital discharge were included in the study.

COVID-19 waves were defined according to hospital directives, which were based on national reports of the daily number of new infections and COVID-19 deaths [14], as well as data from The National Institute of Health Doutor Ricardo Jorge, I.P. (INSA). Throughout the pandemic, INSA monitored the spread of SARS-CoV-2 in Portugal and issued situation reports on the virus’s genetic diversity in collaboration with a nationwide network of hospitals and laboratories [27]. Using this data, SARS-CoV-2 variants were matched with the corresponding waves (Table 1). Patients were then grouped by wave based on their dates of RT-PCR diagnosis or symptom onset, whichever was earlier (this time was considered the disease’s onset date). Due to minor discrepancies of a few days between these two dates, 1.7% of patients were allocated to different waves. Nonetheless, in all cases, patients were assigned to the earliest wave.

Demographic variables included age, age groups (< 60 and ≥ 60 years of age) according to the WHO definition of “elderly,” sex, and geographical area of origin. Other baseline characteristics included patients’ vaccination status (vaccinated or not vaccinated) and the number of days between COVID-19 onset and ICU admission. For vaccinated patients, Pfizer-BioNTech, Astra-Zeneca, Moderna, or Janssen had been administered. Regarding comorbidities, the presence or absence of the following was described: arterial hypertension, diabetes mellitus, dyslipidemia, obesity, chronic respiratory disease, stroke, ischemic heart disease, congestive heart failure, arrhythmias, chronic kidney disease, chronic liver disease, solid cancer, hematologic cancer, hypothyroidism, autoimmune diseases, benign prostatic hyperplasia (BPH), hyperuricemia, acquired immunodeficiency syndrome (AIDS), history of organ transplant, and epilepsy. Chronic respiratory diseases included chronic obstructive pulmonary disease, asthma, and emphysema. Autoimmune diseases included myasthenia gravis, Guillain-Barré syndrome, Crohn’s disease, multiple sclerosis, psoriasis, and rheumatic autoimmune diseases (mainly rheumatoid arthritis). Solid cancers included breast, thyroid, prostate, bladder, colon, and lung cancers, carcinomas, and prostate adenocarcinomas. Hematologic cancers included Hodgkin’s lymphoma, chronic lymphatic leukemia, multiple myeloma, and myeloproliferative syndrome.

Respiratory support concerned the need for invasive mechanical ventilation (IMV), extracorporeal membrane oxygenation (ECMO), and high-flow oxygen (HFO), at least one time during patients’ ICU admission. Admission motives included infection by SARS-CoV-2, emergency surgery, myocardial infarction, stroke, septic shock, kidney failure, and others (which included, for example, arrhythmias and the Guillain–Barre syndrome).

Therapeutics administered at least once during ICU admission were included in the analysis and categorized into different groups, namely antibiotics, antifungal drugs, antiviral drugs, corticosteroids, immunomodulators, and cardiovascular drugs. Cardiovascular drugs were further divided into subcategories, including some of the most commonly used classes: angiotensin receptor antagonists, angiotensin-converting enzyme antagonists, anticoagulants, antiplatelet drugs, beta-blockers, calcium channel antagonists, diuretics, and vasoactive drugs.

Outcome variables included the ICU length of stay, the number of deaths in the ICU (all-cause and from COVID-19), deaths after ICU discharge, days between ICU and hospital discharge, and total deaths. Death in the ICU was analyzed considering the following periods: within 72 h of ICU admission, between 72 h and 7 days after ICU admission, and more than 7 days after ICU admission. However, for the analysis of risk factors associated with death, early mortality (occurring within the first 72 h of ICU admission) and late mortality (after 72 h of ICU admission) were considered.

For the analysis of routine blood analysis results, the maximum values within the first 72 h of ICU admission were obtained for lactate dehydrogenase (LDH), procalcitonin, D-dimer, C-reactive protein (CRP), white blood cell counts (WBCs), and creatinine. For platelet counts, hemoglobin, partial pressure of oxygen (pO2), and lymphocyte counts, the minimum results were obtained.

### Statistical analysis

Categorical data were presented as absolute frequencies and percentages, and continuous variables as median and inter-quartile range (25th percentile; 75th percentile), as they presented asymmetric distributions and deviations from normality. To assess the normality of the continuous variables, Kolmogorov–Smirnov and Shapiro–Wilk tests were used, as appropriate. For comparisons of data between three or more independent groups, such as the six COVID-19 waves, Chi-squared or Fisher’s exact tests were used for nominal variables as appropriate. For continuous variables, the Kruskal–Wallis One-Way ANOVA test was used. Dunn’s post hoc tests were carried out on each pair of groups, using the Bonferroni adjustment in each Dunn’s *p value*.

Time-to-event data was analyzed through event-free survival rates using the Kaplan–Meier estimator. To compare the survival curves corresponding to groups of patients from different waves, the Tarone-Ware test was used.

Additionally, to assess the association between each one of the two outcomes, early and late death, and patient characteristics, clinical data, and laboratory results, an univariable logistic regression analysis was performed. For the multivariable logistic regression model, when it came to patient characteristics and clinical data, solely variables that could be collected at ICU admission were considered. Apart from this exception, all the variables with a *p value* less than 0.25 in the univariable analysis and with clinical relevance were considered. Independent variables were then evaluated for their predictive power, over and above that offered by all the other independent variables. Multivariable models were obtained by a forward stepwise selection method, with the final models corresponding to those with the best area under the receiver operating characteristic (ROC) curve (AUC), among several alternatives. Model performance was assessed using the Hosmer–Lemeshow goodness of fit test, and the AUC was also used to evaluate the discriminative ability of the adjusted models (between discharged and deceased patient groups). Adjusted odds ratio estimates $(\text{a}\widehat{OR})$ and corresponding 95% confidence intervals (95% CI) were obtained for all the variables in the multivariable models.

Statistical analyses were performed using the IBM SPSS Statistics software, version 26 (IBM Corp., New York, USA) and RStudio, version 2022.12.0 (PBC, Boston, USA). The level of significance *α* = 0.05 was considered.

## Results

Data from 1041 patients was analyzed and compared between the six waves of the pandemic, in Portugal. Most of the patients were admitted to the ICU during wave number three (*n* = 295), following waves two (*n* = 186), four (*n* = 162), one (*n* = 135), five (*n* = 132), and six (*n* = 131). Regarding demographics (Table 2), the patients’ age was significantly different between waves (*p* < 0.001). Except for the fourth wave, over half of the patients in each wave were aged 60 or older.

**Table 2:** Patients’ demographics and other baseline characteristics for the six COVID-19 waves

Variables	Wave						
	First (*n* = 135)	Second (*n* = 186)	Third (*n* = 295)	Fourth (*n* = 162)	Fifth (*n* = 132)	Sixth (*n* = 131)	*p* value
Age, years	67.0 (53.0–76.0)	66.0 (54.8–74.3)	61.0 (52.0–70.0)	49.0 (36.8–65.0)	62.5 (51.3–74.0)	69.0 (60.0–78.0)	< 0.001*
Age groups							
< 60	43 (31.9)	64 (34.6)	136 (46.1)	108 (66.7)	51 (38.6)	30 (22.9)	< 0.001
≥ 60	92 (68.1)	122 (65.6)	159 (53.9)	54 (33.3)	81 (61.4)	101 (77.1)	
Sex							
Female	28 (20.7)	56 (30.1)	104 (35.3)	48 (29.6)	41 (31.1)	45 (34.4)	0.074
Male	107 (79.3)	130 (69.9)	191 (64.7)	114 (70.4)	91 (68.9)	86 (65.6)	
Geographical area of origin							
Portugal	91 (67.4)	144 (77.4)	214 (72.5)	89 (54.9)	101 (76.5)	118 (90.1)	< 0.001^**a)**^
Other European countries	1 (0.7)	3 (1.6)	7 (2.4)	14 (8.6)	6 (4.5)	8 (6.1)	
Africa	30 (22.2)	28 (15.1)	37 (12.5)	16 (9.9)	13 (9.8)	2 (1.5)	
Asia	10 (7.4)	8 (4.3)	11 (3.7)	33 (20.4)	4 (3.0)	3 (2.3)	
America	3 (2.2)	3 (1.6)	26 (8.8)	10 (6.2)	8 (6.1)	0 (0.0)	
Vaccine against SARS-CoV-2							
Yes	0 (0.0)	0 (0.0)	0 (0.0)	63 (38.9)	83 (62.9)	101 (77.1)	< 0.001^**b)**^
No	135 (100.0)	186 (100.0)	295 (100.0)	99 (61.1)	49 (37.1)	30 (22.9)	
Days between disease onset and ICU admission	8.0 (6.0–12.0)	8.0 (5.0–11.0)	8.0 (5.5–11.5)	9.0 (6.0–12.0)	5.0 (1.0–10.0)	1.0 (0.0–3.0)	< 0.001*

*p values obtained from the Kruskal–Wallis test; remaining *p* values obtained from the Chi-squared test/Fisher’s exact test

^**a)**^*p* value obtained when comparing patients whose geographical area of origin was Portugal with those from the remaining geographical areas

^**b)**^*p* value obtained from data of waves four to six

**Fig. 1:**
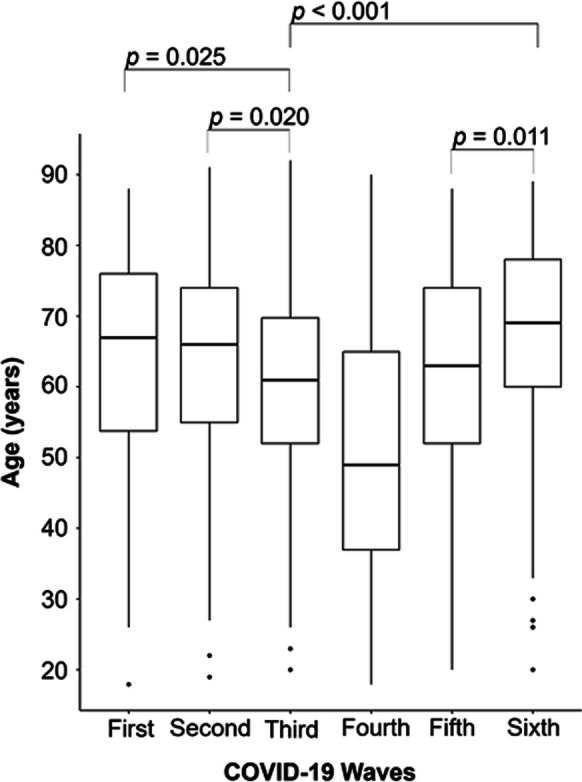
Boxplots for age, by wave. *p* values for the significant differences are presented. *Comparisons with wave four were not presented since the patients were significantly younger than the ones from the remaining waves (all *p* < 0.001)

Patients from the fourth wave were significantly younger (all *p* < 0.001) and had the lowest percentage of elderly individuals (33.3%). In contrast, patients from the sixth wave had the highest percentage of elderly and were significantly older compared to those from waves three and five (*p* < 0.001 and *p* = 0.011, respectively). Additionally, patients from the third wave were significantly younger than those from the first and second waves (*p* = 0.025 and *p* = 0.020, respectively) (Fig. 1).

Despite a higher frequency of men in each one of the waves, there were no significant differences concerning sex. In terms of geographical origin, the majority of the patients in all waves were from Portugal. One interesting aspect is the drop in the percentage of African patients as the pandemic progressed and the significant spike of Asian patients in the fourth wave (Table 2). Although Portugal’s immunization program began on December 27, 2020, only patients from the fourth to sixth waves were vaccinated in this study. After the fifth wave, more than half the patients were already vaccinated. The distribution by vaccine type was quite uniform (*p* = 0.135), despite Pfizer being more frequent (see Supplementary Table 1). The median time from disease onset to ICU admission showed a decreasing trend as the waves progressed, except for wave four (Table 2). Patients in the sixth wave were admitted to the ICU significantly sooner after disease onset compared to all other waves (all *p* < 0.001), followed by those in the fifth wave (all *p* < 0.05) (see Supplementary Fig. 1). This pattern was observed in patients admitted to the ICU both for SARS-Cov-2 infection and for other admission motives (see Supplementary Table 2).

Approximately 83% of patients had at least one comorbidity. The highest percentages of patients with comorbidities were observed in the fifth (91.7%) and sixth (90.8%) waves, while the lowest was in the fourth wave (64.2%). In the second wave, the highest percentages of some of the most common comorbidities were recorded, including arterial hypertension (61.3%), diabetes (39.2%), dyslipidemia (28.0%), and chronic respiratory diseases (14.0%). In contrast, the fifth and sixth waves had higher percentages of less common comorbidities such as stroke, solid or hematologic cancers, history of organ transplant, and autoimmune diseases (Table 3).

**Table 3:** Patients’ comorbidities for the six COVID-19 waves

Comorbidities	Wave						
	First	Second	Third	Fourth	Fifth	Sixth	*p* value
**Yes**	115 (85.2)	156 (83.9)	247 (83.7)	104 (64.2)	121 (91.7)	119 (90.8)	< 0.001
**No**	20 (14.8)	30 (16.1)	48 (16.3)	58 (35.8)	11 (8.3)	12 (9.2)	
Arterial Hypertension	79 (58.5)	114 (61.3)	164 (55.6)	57 (35.2)	64 (48.5)	72 (55.0)	< 0.001
Diabetes	44 (32.4)	73 (39.2)	92 (31.2)	27 (16.7)	40 (30.3)	38 (29.0)	< 0.001
Dyslipidemia	28 (20.7)	52 (28.0)	71 (24.1)	26 (16.0)	31 (23.5)	33 (25.2)	0.162
Obesity	25 (18.5)	34 (18.3)	79 (26.8)	44 (27.2)	24 (18.2)	19 (14.5)	0.013
Chronic respiratory disease	17 (12.6)	26 (14.0)	38 (12.9)	13 (8.0)	18 (13.6)	16 (12.2)	0.615
Stroke	3 (2.2)	6 (3.2)	10 (3.4)	6 (3.7)	9 (6.8)	13 (9.9)	0.016
Ischemic heart disease	11 (8.1)	8 (4.3)	28 (9.5)	9 (5.6)	23 (17.4)	18 (13.7)	< 0.001
Congestive heart failure	5 (3.7)	2 (1.1)	1 (0.3)	3 (1.9)	3 (2.3)	14 (10.7)	-
Arrhythmias	8 (5.9)	12 (6.5)	12 (4.1)	10 (6.2)	8 (6.1)	25 (19.1)	< 0.001
Chronic kidney disease	14 (10.4)	12 (6.5)	17 (5.8)	8 (4.9)	17 (12.9)	19 (14.5)	0.005
Chronic liver disease	4 (3.0)	2 (1.1)	5 (1.7)	3 (1.9)	7 (5.3)	4 (3.1)	-
Solid Cancer	11 (8.1)	6 (3.2)	17 (5.8)	4 (2.5)	13 (9.8)	17 (13.0)	0.001
Hematologic cancer	5 (3.7)	5 (2.7)	7 (2.4)	5 (3.1)	13 (9.8)	10 (7.6)	0.003
Hypothyroidism	2 (1.5)	7 (3.8)	18 (6.1)	6 (3.7)	5 (3.8)	7 (5.3)	0.347
Autoimmune disease	3 (2.2)	8 (4.3)	9 (3.1)	9 (5.6)	9 (6.8)	6 (4.6)	0.388
BPH	9 (6.6)	10 (5.4)	8 (2.7)	4 (2.5)	2 (1.5)	6 (4.6)	0.143
Hyperuricemia	13 (9.6)	0 (0.0)	7 (2.4)	6 (3.7)	5 (3.8)	6 (4.6)	-
AIDS	3 (2.2)	0 (0.0)	12 (4.1)	2 (1.2)	3 (2.3)	2 (1.5)	-
History of organ transplant	4 (2.9)	6 (3.2)	7 (2.4)	1 (0.6)	13 (9.8)	5 (3.8)	-
Epilepsy	0 (0.0)	2 (1.1)	6 (2.0)	2 (1.2)	5 (3.8)	4 (3.1)	-

All *p* values were obtained by Chi-squared test/Fisher’s exact test. *p* values for variables which frequencies were too low/null were not reported. *BPH* benign prostatic hyperplasia, *AIDS* acquired immunodeficiency syndrome

The primary reason for ICU admission was “Infection by SARS-CoV-2” (86.6% of all patients), which accounted for more than 90% of admissions from wave one to four. Beyond that point, admissions due to SARS-CoV-2 infection decreased, while other causes such as emergency surgery, stroke, septic shock, and myocardial infarction became more frequent (Table 4).

**Table 4:** Patients’ admission motives for the six COVID-19 waves

Variables	Wave					
	First (*n* = 135)	Second (*n* = 186)	Third (*n* = 295)	Fourth (*n* = 162)	Fifth (*n* = 132)	Sixth (*n* = 131)
Infection by SARS-CoV-2	125 (92.6)	176 (94.6)	280 (94.9)	147 (90.7)	92 (69.7)	50 (38.2)
Emergency surgery	7 (5.2)	1 (0.5)	3 (1.0)	4 (2.5)	14 (10.6)	15 (11.5)
Myocardial infarction	0 (0.0)	2 (1.1)	3 (1.0)	1 (0.6)	5 (3.8)	12 (9.2)
Stroke	0 (0.0)	3 (1.6)	4 (1.4)	1 (0.6)	5 (3.8)	25 (19.1)
Septic shock	0 (0.0)	2 (1.1)	0 (0.0)	5 (3.1)	7 (5.3)	16 (12.2)
Kidney failure	0 (0.0)	1 (0.5)	1 (0.3)	2 (1.2)	3 (2.3)	4 (3.1)
Other	3 (2.2)	1 (0.5)	4 (1.3)	2 (1.2)	6 (4.6)	9 (6.8)

**Fig. 2:**
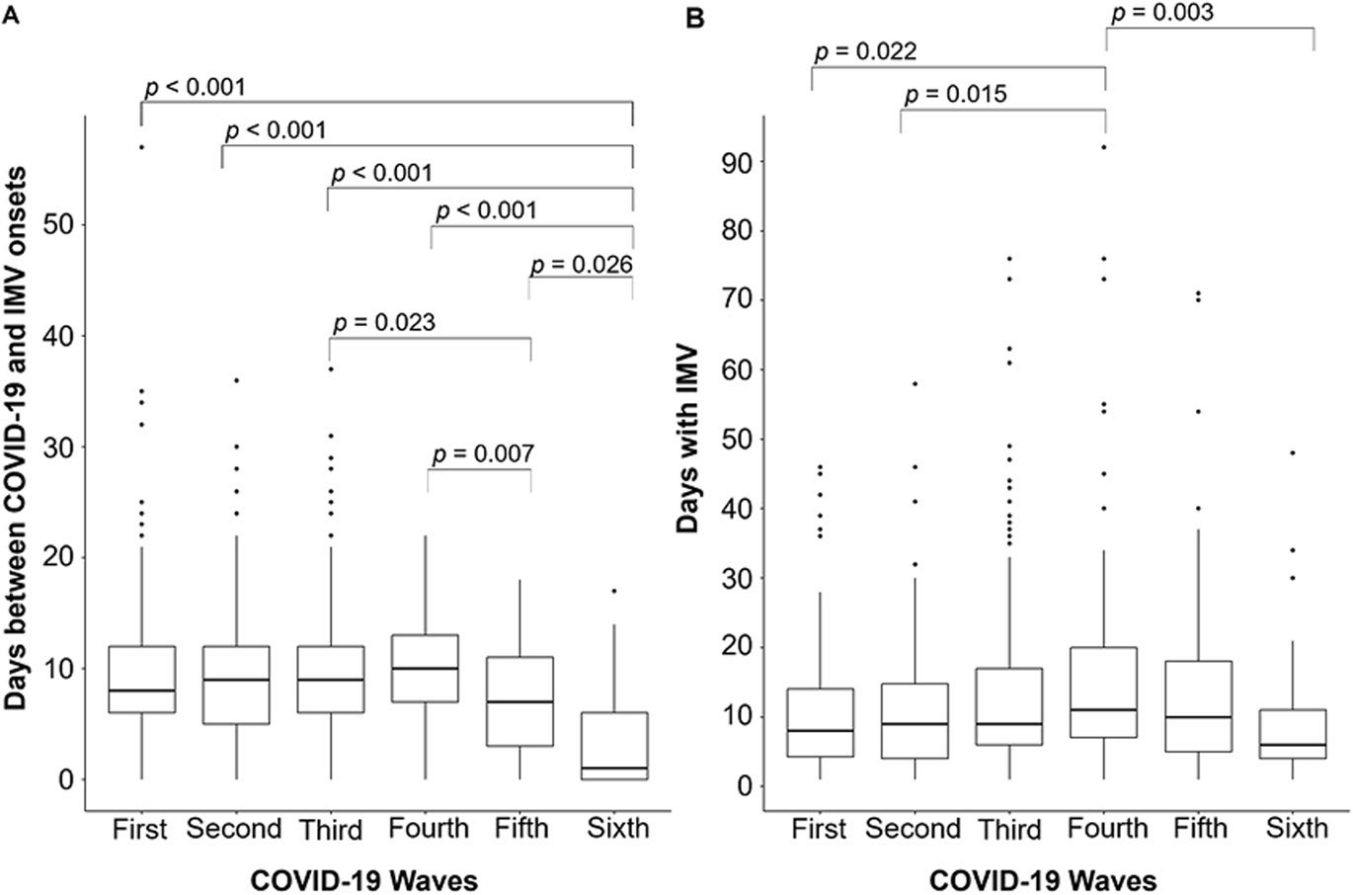
Boxplots for the number of days between disease and IMV onsets (**A**) and the number of days patients spent on IMV (**B**), by wave, and significant results from multiple comparison tests between them

Regarding respiratory support, the third wave had the greatest percentage of patients requiring IMV (82.4%), whereas the fourth wave had the largest percentage of patients requiring ECMO (14.8%) (Table 5). It is important to note that all patients who required IMV and/or ECMO were admitted specifically due to SARS-CoV-2 infection. The median time between disease and IMV onset was significantly shorter in the sixth wave (all *p* < 0.05) (Fig. 2A). Besides, patients from the fourth wave spent significantly more time on IMV compared to those in the first (*p* = 0.022), second (*p* = 0.015), and sixth waves (*p* = 0.003) (Fig. 2B). Furthermore, a significant negative correlation between the need for IMV and HFO was observed across all waves, with the strongest correlations occurring in the first four waves (φ = − 0.681, − 0.872, − 0.806, and − 0.749, respectively) (see Supplementary Table 3).

**Table 5:** Respiratory support techniques by wave

Variables	Wave						
	First (*n* = 135)	Second (*n* = 186)	Third (*n* = 295)	Fourth (*n* = 162)	Fifth (*n* = 132)	Sixth (*n* = 131)	*p* value
IMV	110 (81.5)	131 (70.4)	243 (82.4)	109 (67.3)	72 (54.5)	37 (28.2)	< 0.001
Days between disease and IMV onsets	8.5 (6.0–13.0)	10.0 (5.0–12.0)	9.0 (6.0–12.0)	10.0 (7.0–13.0)	7.0 (2.5–11.5)	1.0 (0.0–6.0)	< 0.001*
Days with IMV	8.0 (4.0–14.3)	9.00 (4.0–15.0)	10.0 (6.0–17.0)	11.0 (7.0–20.0)	11.0 (5.0–18.0)	6.0 (3.5–12.0)	< 0.001*
ECMO	14 (10.4)	18 (9.7)	21 (7.1)	24 (14.8)	11 (8.3)	1 (0.8)	0.001
Days between disease and ECMO onsets	12.5 (10.0–17.0)	13.0 (8.8–16.3)	15.0 (13.0–18.5)	17.0 (12.5–21.0)	13.0 (3.0–14.0)	15.0 (15.0–15.0)	0.033*
Days with ECMO	9.5 (4.0–20.0)	13.5 (5.0–31.3)	15.0 (7.0–37.0)	14.5 (6.0–21.8)	8.0 (2.0–26.0)	7.0 (7.0–7.0)	0.680*
HFO	15 (11.1)	45 (24.2)	36 (12.2)	37 (22.8)	20 (15.0)	12 (9.2)	< 0.001

*p values were obtained from the Kruskal–Wallis test; remaining *p* values were obtained from the Chi-squared test/Fisher’s exact test. *IMV* invasive mechanical ventilation, *ECMO* extracorporeal membrane oxygenation, *HFO* high-flow oxygen

In Table 6, all therapeutics administered during the six waves are presented, rather than focusing on those prescribed specifically for COVID-19, as several patients had multiple comorbidities that required different treatments. Performing such separation would be challenging due to the overlapping treatment regimens. Therefore, all medication was considered in order to better understand how the distribution of each therapeutic group varied across different waves and to explore any potential relationship with disease severity.

**Table 6:** Patients’ administered therapeutics during ICU stay, by wave

Variables	Wave						
	First (*n* = 134)	Second (*n* = 182)	Third (*n* = 286)	Fourth (*n* = 159)	Fifth (*n* = 124)	Sixth (*n* = 124)	*p* value
Antibiotics	110 (82.1)	137 (75.3)	243 (85.0)	122 (76.7)	103 (83.1)	90 (72.6)	0.021
Antifungal drugs	41 (30.6)	38 (20.9)	90 (31.5)	36 (22.6)	34 (27.4)	18 (14.5)	0.003
Antiviral drugs	53 (39.6)	92 (50.5)	75 (26.2)	35 (22.0)	22 (17.7)	12 (9.7)	< 0.001
Cardiovascular drugs							
Angiotensin receptor antagonists	20 (14.9)	16 (8.8)	31 (10.8)	7 (4.4)	10 (8.1)	14 (11.3)	0.060
Angiotensin-converting enzyme antagonists	61 (45.5)	73 (40.1)	113 (39.5)	45 (28.3)	50 (40.3)	53 (42.7)	0.052
Anticoagulants	130 (97.0)	175 (96.2)	278 (97.2)	156 (98.1)	112 (90.3)	97 (78.2)	< 0.001
Antiplatelet drugs	25 (18.7)	35 (19.2)	56 (19.6)	18 (11.3)	42 (33.9)	45 (36.3)	< 0.001
Beta-blockers	47 (35.1)	56 (30.8)	96 (33.6)	45 (28.3)	61 (49.2)	55 (44.4)	0.001
Calcium channel antagonists	45 (33.6)	36 (19.8)	68 (23.8)	27 (17.0)	38 (30.6)	35 (28.2)	0.006
Diuretics	101 (75.4)	131 (72.0)	212 (74.1)	100 (62.9)	90 (72.6)	67 (54.0)	< 0.001
Vasoactive drugs	84 (62.7)	111 (61.0)	209 (73.1)	85 (53.5)	81 (65.3)	38 (30.6)	< 0.001
Corticosteroids	120 (89.6)	162 (89.0)	261 (91.3)	151 (95.0)	97 (78.2)	67 (54.0)	< 0.001
Immunomodulators	26 (19.4)	9 (4.9)	10 (3.5)	3 (1.9)	14 (11.3)	5 (4.0)	< 0.001

All *p* values were obtained by Chi-squared test/Fisher’s exact test

The highest percentages of administered medications were observed for anticoagulants, with about 94% of all patients receiving them, followed by corticosteroids at 85%, and antibiotics at 80%. Overall, approximately 98% of patients were prescribed at least one cardiovascular drug. The sixth wave presented the lowest percentages of antibiotic (72.6%), antifungal (14.5%), antiviral (9.7%), anticoagulant (78.2%), and corticosteroid (54.0%) administration. In contrast, the highest percentages of these medications were observed in the first three waves of the pandemic. Remdesivir was the most commonly used antiviral (23.8% of all cases—data not shown), particularly during the second (49.5%), third (24.8%), and first waves (23.9%). Following the fourth wave, the percentage of remdesivir usage declined from 19.5 to 4.0% in the sixth wave. Cardiovascular drugs were also primarily administered in the first three waves, with a notable decline in the fourth and sixth waves.

**Fig. 3:**
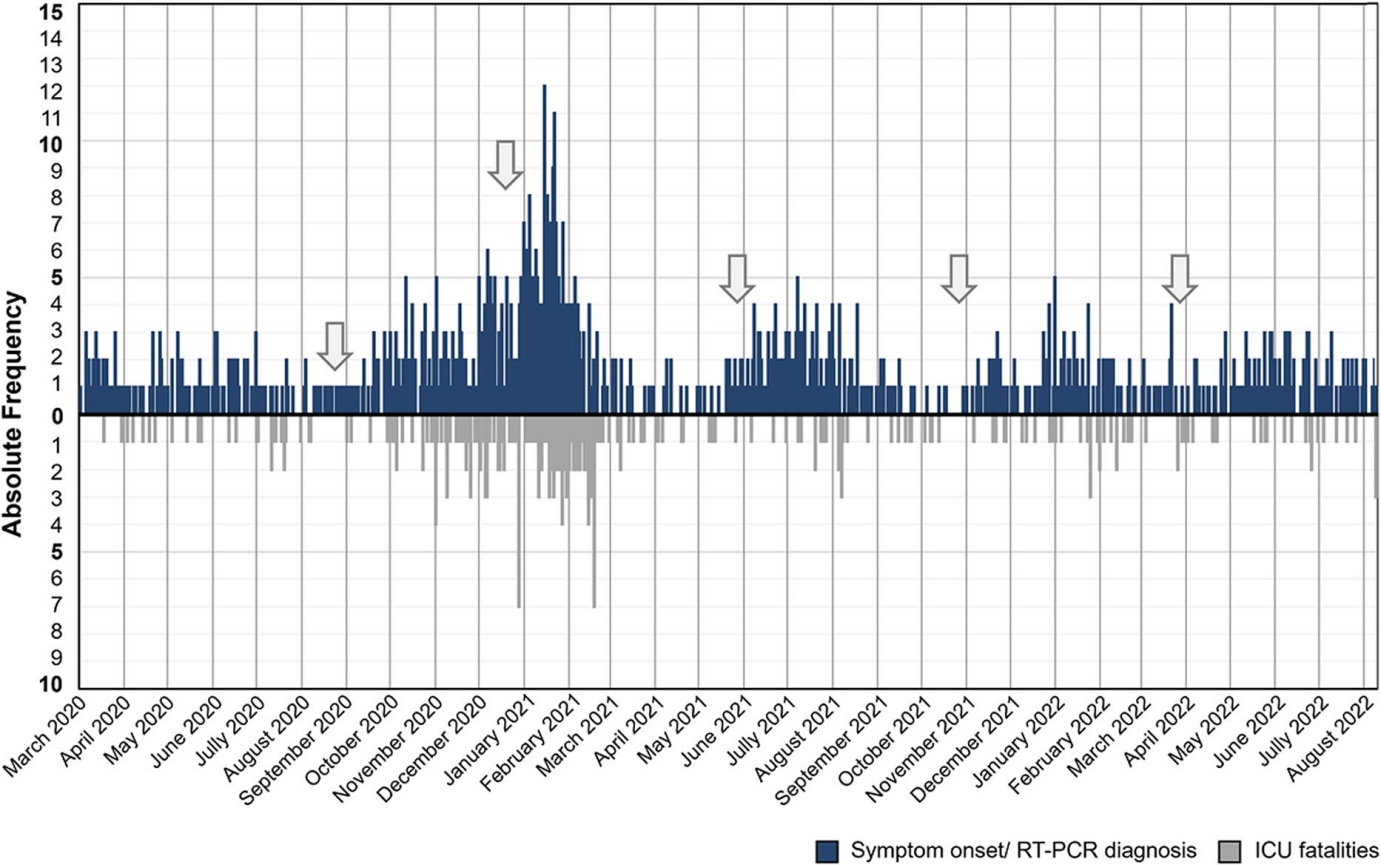
Daily number of patients starting COVID-19 symptoms or with positive RT-PCR tests and fatalities following ICU admission, between March 10, 2020, and August 8, 2022. The arrows mark the end of waves number one, two, three, four, and five, respectively. Vertical lines mark the first day of each month

**Fig. 4:**
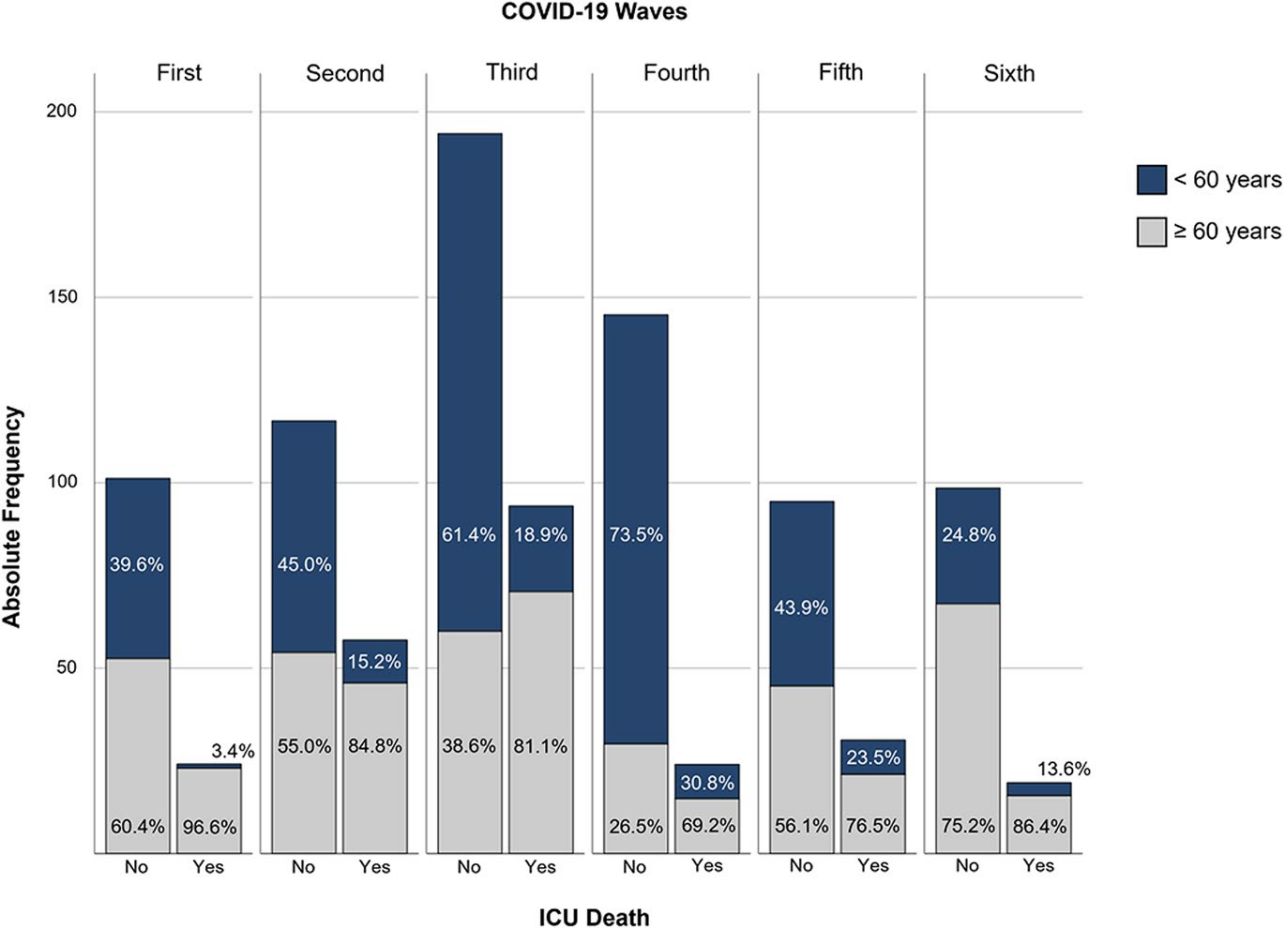
Absolute frequency (*y*-axis) and percentages (within bars) of patients in each age group (< 60 or ≥ 60 years), by the outcome “ICU Death” (yes/no), across waves

Patients’ outcome variables in the ICU and after ICU discharge are presented in Table 7. During the sixth wave, patients had a significantly shorter ICU stay (all *p* < 0.001, see Supplementary Fig. 2). ICU mortality was significantly higher in the third wave (*p* < 0.001) and lowest in the fourth and sixth waves. This trend is highlighted in Fig. 3, showing that from the end of August 2020 to the end of May 2021, there was not only an increase in SARS-CoV-2 infections but also a higher number of ICU deaths. In waves one through five, fatalities were significantly more common among patients aged 60 and older (all *p* < 0.05), with over 70% of ICU deaths occurring in this age group (see Supplementary Table 4 and Fig. 4).

**Table 7:** Patients’ outcomes in the six COVID-19 waves

Variables	Wave						
	First	Second	Third	Fourth	Fifth	Sixth	*p* value
ICU length of stay	10.0 (5.0–16.0), *n* = 135	9.0 (5.0–15.3), *n* = 186	10.0 (5.0–18.0), *n* = 295	9.0 (5.0–16.0), *n* = 162	7.0 (3.0–17.0), *n* = 132	4.0 (3.0–7.0), *n* = 131	< 0.001*
ICU deaths	29/135 (21.5)	66/186 (35.5)	106/295 (35.9)	26/162 (16.0)	34/132 (25.8)	22/131 (16.8)	< 0.001
Proportion of ICU deaths due to COVID-19	29/29 (100.0)	65/66 (98.5)	104/106 (98.1)	22/26 (84.6)	29/34 (85.3)	14/22 (63.6)	NA
Deaths after ICU discharge	8/106 (7.5)	15/120 (12.5)	8/189 (4.2)	6/136 (4.4)	14/98 (14.3)	17/109 (15.6)	0.001
Proportion of deaths after ICU discharge due to COVID-19	7/8 (87.5)	13/15 (86.7)	7/8 (87.5)	4/6 (66.7)	11/14 (78.6)	4/17 (23.5)	NA
Days between ICU and hospital discharge	14.0 (8.0–26.3), *n* = 106	11.0 (7.0–20.5), *n* = 120	13.0 (7.5–26.5), *n* = 189	12.0 (7.0–23.8), *n* = 136	13.0 (5.0–27.3), *n* = 98	15.0 (6.0–28.0), *n* = 109	0.322*
Total deaths	37/135 (27.4)	81/186 (43.5)	114/295 (38.6)	32/162 (19.8)	48/132 (36.4)	39/131 (29.8)	< 0.001

*p values were obtained from the Kruskal–Wallis test; remaining* p* values were obtained from the Chi-squared test/Fisher’s exact test

Considering the cause of ICU death, COVID-19 accounted for nearly 99% of all ICU fatalities during the first three waves. However, this proportion decreased in the subsequent waves, reaching its lowest (63.6%) in the sixth wave (Table 7). After ICU discharge, the highest percentage of deaths was observed in the fifth (14.3%) and the sixth (15.6%) waves. Despite fewer ICU admissions due to SARS-Cov-2 infection during these waves, COVID-19 still accounted for 78.6% of deaths after ICU discharge in the fifth wave. On the other hand, this percentage dropped to its lowest, 23.5%, in the sixth wave (Table 7).

**Fig. 5:**
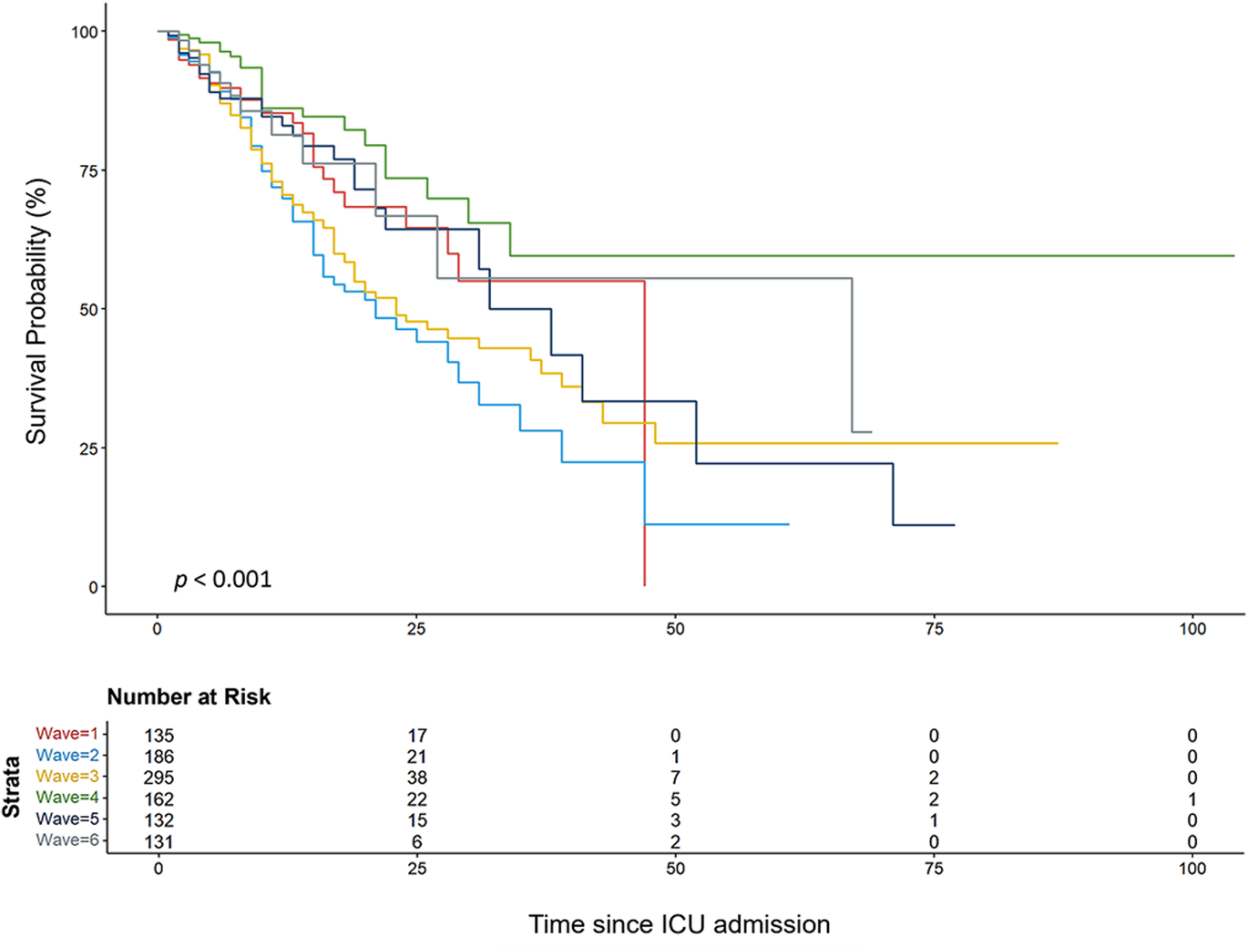
Kaplan–Meier survival estimates for death due to COVID-19 in the ICU, by wave

Considering only patients who died due to COVID-19, the risk of death significantly differed across waves (*p* < 0.001) (Fig. 5). The risk of death was significantly lower in the fourth wave, compared to waves two, three, and five (*p* < 0.001, *p* < 0.001, and *p* = 0.040, respectively). The first wave also exhibited a lower risk compared to the second wave (*p* = 0.040).

Considering death due to COVID-19, an analysis considering the timing of death was also performed (Table 8). A small percentage of patients (approximately 14.8%) died within the first 72 h of ICU admission. Most deaths occurred after 7 days of ICU admission across all waves, with the highest percentages observed in the fourth (72.7%) and second (69.2%) waves.

**Table 8:** Timing of death due to COVID-19, across waves

	Within 72 h of ICU admission	Within 72 h and 7 days of ICU admission	Beyond 7 days of ICU admission
First wave	8 (27.6)	5 (17.2)	16 (55.2)
Second wave	10 (15.4)	10 (15.4)	45 (69.2)
Third wave	10 (9.6)	29 (27.9)	65 (62.5)
Fourth wave	2 (9.1)	4 (18.2)	16 (72.7)
Fifth wave	6 (20.7)	7 (24.1)	16 (55.2)
Sixth wave	3 (21.4)	5 (35.7)	6 (42.9)

**Fig. 6:**
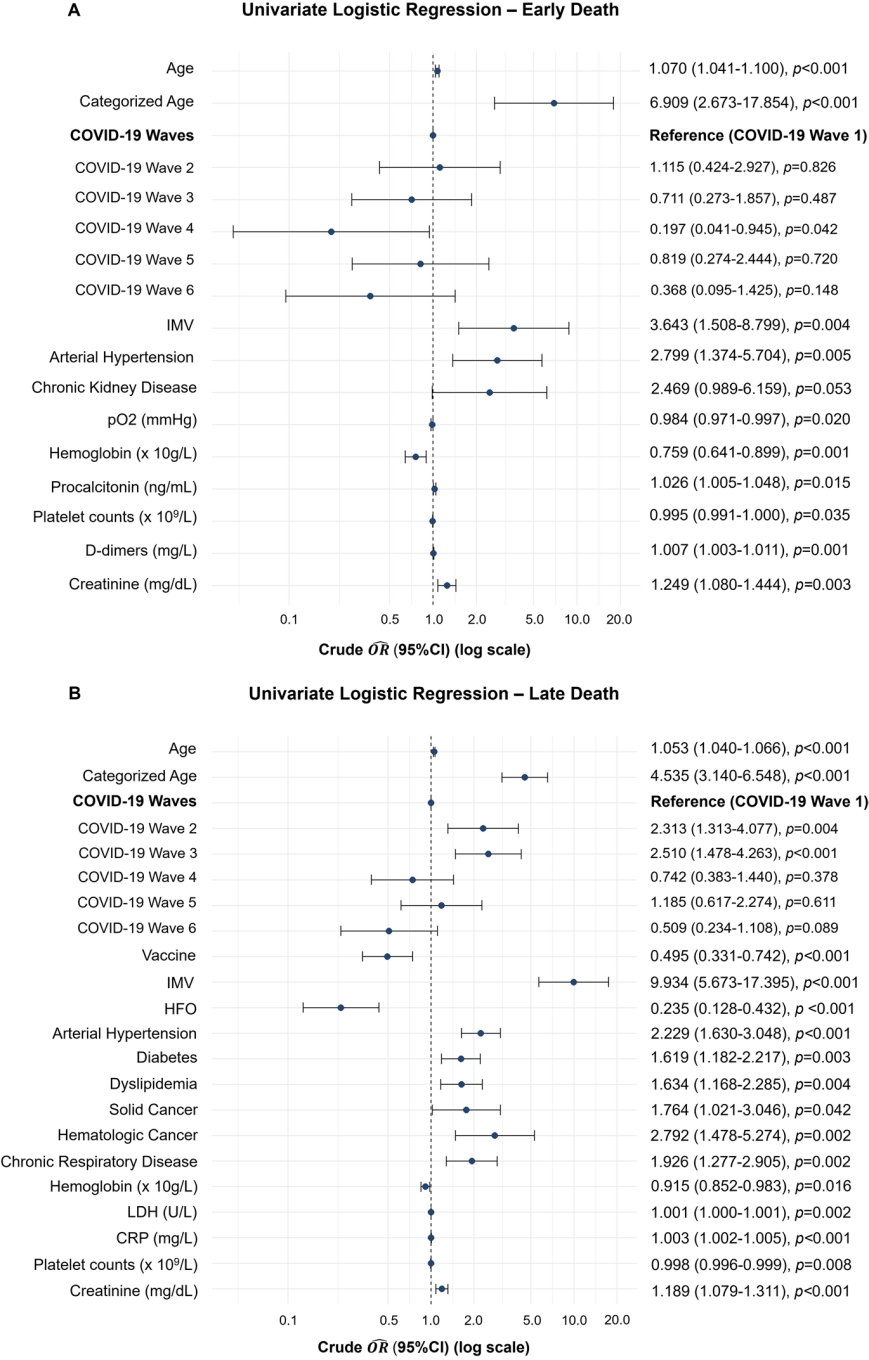
Significant results from the univariate logistic regression for early **A** and late death **B**. *The reference category for the variable “Categorized age” was being 60 or older, and for the variable “COVID-19 Wave” was wave number 1. Abbreviations: $\widehat{OR}$, odds ratios estimates; CI, confidence interval; IMV, invasive mechanical ventilation; pO2, partial pressure of oxygen; HFO, high flow oxygen; LDH, lactate dehydrogenase; CRP, C-reactive protein

To understand the factors associated with the timing of death due to COVID-19, independent risk factors for both early and late mortality were identified. For that, patients were categorized into two groups: those who died within the first 72 h of ICU admission (early mortality), and those who died after 72 h of ICU admission (late mortality). Both groups were characterized, as well as the group of patients that was discharged from the ICU (see Supplementary Table 5). Univariate logistic regression was then performed (see Supplementary Table 6), with the most significant findings highlighted in Fig. 6. Patients aged 60 or older had a significantly higher risk of death, approximately 7 times higher for early death and 5 times higher for late death. Compared to patients from the first wave, those from the second ($\widehat{OR}$ 2.313; 95% CI, 1.313–4.077) and third ($\widehat{OR}$ 2.510; 95% CI, 1.478–4.263) waves presented a significantly higher risk of late death. Interestingly, vaccination was only associated with a lower risk of death in patients who died after the first 72 h of ICU admission ($\widehat{OR}$ 0.495; 95% CI, 0.331–0.742). IMV increased the risk of both early ($\widehat{OR}$ 3.643; 95% CI, 1.508–8.799) and late ($\widehat{OR}$ 9.934; 95% CI, 5.673–17.395) death, while HFO was linked to a lower risk of late death ($\widehat{OR}$ 0.235; 95% CI, 0.128–0.432). Among comorbidities, only arterial hypertension significantly increased the risk of early death. However, for late death, additional factors such as dyslipidemia, diabetes, chronic respiratory diseases, and solid or hematologic cancers were all associated with an increased risk.

**Fig. 7:**
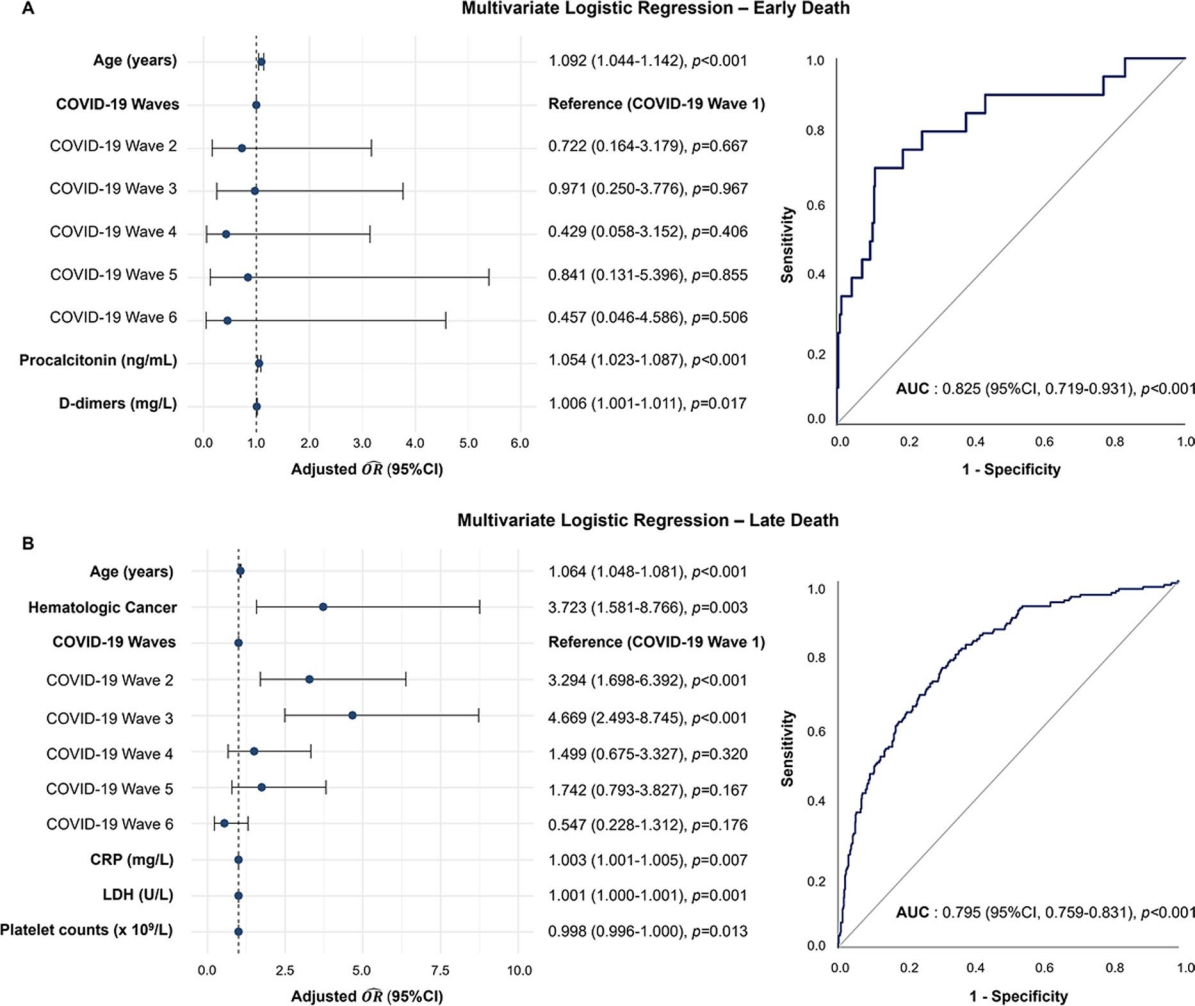
Results from the multivariable logistic regression for early (**A**) and late death (**B**). Abbreviations: $\widehat{OR}$, odds ratio estimates; CI, confidence interval; LDH, lactate dehydrogenase; CRP, C-reactive protein; AUC, area under the receiver operating characteristic curve

To develop the multivariate logistic regression models, only independent risk factors identified in the univariate analysis that could be measured within the first 72 h of ICU admission were used, to allow the assessment of patients’ prognosis upon ICU admission. The results demonstrated that age significantly impacted patient outcomes in both early (a $\widehat{OR}$ 1.092; 95% CI, 1.044–1.142) (Fig. 7A) and late (a $\widehat{OR}$ 1.064; 95% CI, 1.048–1.081) (Fig. 7B) death. Both models were adjusted for COVID-19 waves, but only in the case of late death was this factor a significant contributor to the patients’ outcomes. Specifically, patients from the second and third waves presented a higher risk of death in comparison to those from the first wave, with the risk being approximately three times higher in the second wave and five times higher in the third. Just one comorbidity, namely hematologic cancer, was significantly associated with an increased risk of late mortality (a $\widehat{OR}$ 3.723; 95% CI, 1.581–8.766). Regarding laboratory biomarkers, higher levels of procalcitonin (a $\widehat{OR}$ 1.054; 95% CI, 1.023–1.087) and D-dimers (a $\widehat{OR}$ 1.006; 95% CI, 1.001–1.011) were associated with a greater risk of early death. For late death, patients with higher levels of CRP (a $\widehat{OR}$ 1.003; 95% CI, 1.001–1.005) and LDH (a $\widehat{OR}$ 1.001; 95% CI, 1.000–1.001) were at significantly higher risk, whereas higher platelet counts indicated a lower risk (a $\widehat{OR}$ 0.998; 95% CI, 0.996–1.000).

## Discussion

This study offers an in-depth analysis and comparison of COVID-19 patient characteristics across the six pandemic waves in Portugal. As indicated by our preliminary research, once again we concluded that accounting for COVID-19 waves is crucial when conducting such analyses [28]. Thus, we highlighted the impact of the different variants and the effect of Portugal’s immunization program on the characteristics and outcomes of ICU patients. Furthermore, to identify the factors associated with both early and late mortality due to COVID-19, laboratory analyses from the first 72 h of ICU admission were examined. Using the mentioned laboratory data, along with patients’ demographic and clinical characteristics, two logistic regression models were developed for both early and late mortality, with AUC values from ROC curve analyses of 0.825 (*p* < 0.001; 95% CI, 0.719–0.931) and 0.795 (*p* < 0.001; 95% CI, 0.759–0.831), respectively.

Main demographic findings indicated a significant decrease in the median age of patients admitted to the ICU during the fourth wave, followed by an increase in the last two waves. This may be related to the implementation of the Portuguese immunization program at the beginning of the third wave, which was primarily directed to people over the age of 50 and with certain underlying health conditions [29]. Consequentially, a slight decrease in patients’ age was already observed by the end of the third wave (which recorded the second lowest median age), followed by an even more significant decrease during Portugal’s fourth wave, which was associated with the Delta variant. At this time, besides a great part of the elderly population being fully vaccinated, the implementation of immunization programs led to the abolition of some preventive measures against the populational spread of SARS-CoV-2 (alongside, e.g., reopening of nurseries, schools, sports, and restaurants), which may have contributed to more cases of infection among younger individuals. Thus, the older population appeared to be less affected by SARS-Cov-2 than the younger, unvaccinated part of the population, highlighting the effectiveness of vaccination in controlling the transmission of the Delta variant [24]. In fact, the lowest ICU mortality for patients over 60 years of age was observed during the fourth wave (69.2%), while the highest mortality for those under 60 was also recorded at that time (30.8%). Following this event, by the mid-to-late fourth wave, most of the population had received at least one dose of the vaccine due to the accelerated implementation of the immunization plan. As a result, the median age of patients increased, returning to values closer to those observed during the first and second waves, as well as ICU mortality for those over 60 years of age. In various studies, mortality has been shown to be greater in older patients, which has been linked to the increased immune system disfunction, the larger number of comorbidities, and the higher likelihood of altered mental status with disorientation and/or delirium [30]. Thus, despite a higher percentage of deaths among patients under 60 years of age during the fourth wave, this wave also presented the lowest mortality risk, reflecting the protective effect of age [31].

In the fifth and sixth waves of the pandemic, when the omicron lineages were prominent and vaccination was widespread in Portugal, the number of COVID-19 cases decreased significantly [32]. A corresponding decrease in the use of antivirals, such as remdesivir, was observed. Remdesivir, the first COVID-19 medication authorized by the European union and approved in Portugal in Jully 2020, was primarily used for patients over 12 years of age with severe disease, and who required supplemental oxygen [33]. Initially, it demonstrated significant antiviral proprieties and clinical benefits, such as reduced pulmonary infiltrates; however, as the pandemic progressed, different studies revealed that remdesivir did not consistently provide significant clinical improvements in the outcomes of COVID-19 patients [34, 35]. At this time, national testing strategies were well established, and the government maintained preventive contingency plans that included specific holiday restrictions (e.g., during Christmas 2021) to prevent the spread of disease. This relieved the health-care system, resulting in an increase in ICU admissions for reasons other than infection by SARS-CoV-2. Similar trends were observed in other countries, e.g., Spain, particularly during the fifth wave [36]. As a result, in the ICUs of ULSSJ, the time from disease onset to ICU admission was shortened, not only due to the less crowded ICUs, but also because some patients were diagnosed with COVID-19 after being admitted for other reasons or emergencies. This likely contributed to the observed decrease in the time between disease onset and the initiation of IMV. Comparatively, in Malawi, there was a significant reduction in the median number of days between COVID-19 symptom onset and ICU admission as the pandemic evolved [37]. This trend may reflect an overall improvement in the efficiency of patient management during the global health crisis, as demonstrated by decreasing mortality and shorter ICU admission times, especially after the third wave [38–40].

Regarding respiratory support, a great percentage of patients required IMV in the first three waves, with rates consistently above 70%. While other reports have shown trends similar to those in this study, IMV percentages were noticeably lower. For example, a comprehensive comparison of the first five COVID-19 waves in Spain found that IMV was significantly more required in the first wave (13.5%) compared to the second (5.5%), third (7.0%), and fifth (3.0%) waves [36]. Other reports have also documented a comparable pattern of decreased need for IMV from the first to the second waves [40–42], with some even showing a subsequent increase in the third wave [36, 38], as observed in the present case. This could have been related to the implementation of alternative/complementary respiratory support techniques such as HFO, which may have reduced the need for IMV in certain waves. This was supported by the significant inverse correlation between the two variables, suggesting that one method might have been preferred over the other depending on the patients’ characteristics and disease severity. In fact, studies have shown that the early use of HFO can reduce the need for IMV, shortening clinical recovery, and thereby reducing the burden on ICUs caused by the extended time patients spend on IMV [43, 44]. Following the third wave, a higher percentage of patients aged 60 or younger required IMV compared to those over 60, likely due to the decrease in median patient age. Nonetheless, the overall need for IMV and other respiratory support techniques progressively decreased, possibly reflecting the reduced severity of the disease in subsequent waves.

Indeed, the highest percentages of ICU mortality and risk of death were observed during the second and third waves when the alpha variant was predominant, and the lowest during the fourth and sixth waves. Other studies have similarly documented that the alpha phase had a higher ICU death rate compared to the delta period (i.e., fourth wave in Portugal), largely because the elderly were more affected, and the population was not yet vaccinated [25]. When the omicron variant emerged, mortality decreased even further, as expected, due to the virus’s much lower virulence and the public health measures already in place. For instance, a study conducted in the Netherlands, which involved a much larger sample size of 18,772 patients admitted to different ICUs across the country, showed reduced odds ratios for hospital mortality during the omicron phase [45]. Thus, the much higher virulence observed in the first waves significantly impacted the frailer part of the population, leading to a greater need for critical care. Despite being included in the pre-vaccination period, the first wave had one of the lowest percentages of death. This could be related to Portugal’s early implementation of economic and social restrictions, as well as strict containment measures. The elevated level of compliance by the Portuguese population strongly impacted the daily number of deaths in the country, as well as the number of occupied ICU beds, compared to what was predicted [46]. As a result, in Portugal, only 5.8% of the total fatalities registered in 2020 were related to COVID-19 [47].

When it comes to the timing of death, only a limited number of studies have specifically examined this factor within the ICU setting [48, 49]. The majority of research has focused on the timing of death throughout the hospitalization period (e.g., within the first 2 weeks of hospitalization, versus after 2 weeks)[50], as well as after hospital discharge [51–54]. Considering death within the first 72 h of ICU admission, a case–control study involving 261 patients from Ethiopia reported that 31.8% of deaths occurred during this period [49]. In comparison, our study found that 14.8% of COVID-19-related deaths took place during the same early period. Despite these relatively high percentages, the majority of deaths occurred beyond the 72 h in both studies. This fact could be related to the admission of patients in the need for extended ICU care, such as those with refractory respiratory failure requiring prolonged IMV. These patients face a greater risk of developing ICU-acquired pneumonia and bacterial co-infections, further increasing their risk of death later in their ICU stay [55, 56]. This susceptibility to secondary infections extends beyond patients needing IMV and includes those requiring other invasive therapies such as ECMO and hemodialysis. Furthermore, the presence of comorbidities increases the risk of a poor prognosis, as these patients have less capacity to withstand and recover from injury, potentially resulting in persisting COVID-19-related symptoms, often driven by a non-resolving inflammatory response [57].

Several comorbidities have already been recognized as significant factors in the course and outcome of COVID-19 [51, 57]; however, their impact on mortality within the specific timeframes selected for this study has not yet been studied in dept. Using logistic regression, we identified arterial hypertension and chronic kidney disease as significant risk factors for early death in the univariate analysis. Notably, a study that employed machine learning techniques on data from over two million COVID-19 patients across 146 countries also identified hypertension and chronic kidney disease as key factors strongly correlated with patients’ mortality risk [58]. Arterial hypertension has frequently been linked to severe and even fatal outcomes in COVID-19 patients. A possible explanation that has been widely discussed is that hypertensive patients have elevated angiotensin-converting enzyme (ACE) 2 expression, the receptor that SARS-CoV-2 uses to enter lung cells, due to the use of ACE inhibitors or angiotensin receptor blockers to manage blood pressure [59, 60]. Coupled with the associated chronic inflammation and endothelial dysfunction often linked to hypertension, this helps explain the link between this comorbidity and its contribution to both early and late mortality risks in ICU patients. For late death, several other comorbidities, namely diabetes, dyslipidemia, and both solid and hematologic cancers, were associated with an increased risk of mortality. While the associations of the mentioned comorbidities with COVID-19 are well established [57, 59, 60], after adjusting for potential confounders in the multivariate analysis, only specific laboratory biomarkers and age remained associated with an increased risk of death, with the exception of hematologic cancer. The association between this comorbidity and late death is evident, as individuals with compromised immune systems, either due to cancer treatments, adjuvant steroid therapy, or disease predisposition, are at increased risk not only of COVID-19 infection but also of higher mortality after infection [60, 61]. Moreover, the potential link between infection by SARS-CoV-2 and cancer pathophysiology, through the activation of inflammatory pathways culminating in a cytokine storm, is a major contributor to multiorgan failure in COVID-19 patients, further worsening cancer patients’ prognosis. Due to these associations, subsets of even greater risk include patients with lung cancer and hematological malignancies [60]. For example, in a study comparing the outcomes of 463 patients with solid tumors and 463 with hematological cancers, those with hematological malignancies experienced more severe disease, required more IMV, had longer hospital stays, and showed a higher case fatality rate (14.9% vs. 4.8% in solid cancers) [61]. Furthermore, in a meta-analysis that included data from 12,057 patients, the crude OR for all-cause mortality was 1.64 higher for patients with hematological cancer, in comparison to solid cancer [62]. All this evidence further underscores the significance of hematological cancer in the obtained multivariable regression model, in comparison to solid cancers that only showed relevance in the univariate analysis.

Finally, considering the laboratory analysis results, different biomarkers were linked to mortality at the two studied timings, suggesting that each is influenced by distinct factors. To the best of our knowledge, no other studies have evaluated the different biomarkers associated with COVID-19 mortality at the selected timepoints during ICU admission. Higher levels of procalcitonin and D-dimers were associated with a higher risk of early mortality, while late mortality was linked to higher levels of CRP and LDH, along with lower platelet counts. These same markers have been considered extremely useful for identifying COVID-19 patients at higher risk of death, especially when analyzed in multivariable models [63–65]. Procalcitonin is primarily a marker of bacterial infection [66], meaning that the presence of concurrent bacterial infections in COVID-19 patients early in their ICU admission could contribute to elevated levels of this biomarker, highlighting its significance in predicting early death in the obtained multivariable model. However, several recent studies have discarded this option, demonstrating that procalcitonin is a poor indicator of bacterial co-infection in COVID-19 patients, as its levels can also rise due to inflammation or excessive cytokine production. Nevertheless, this biomarker remains valuable for predicting disease severity and adverse outcomes in COVID-19 [67–69]. D-dimers, biomarkers of coagulation and fibrinolysis, are closely related to microvascular thrombotic processes in COVID-19. Additionally, they are also related to the inflammatory responses that accompany the disease. Hence, elevated D-dimer levels have been linked not only to generalized coagulopathy, but also to organ disfunction and respiratory failure, acting as predictors of disease severity, progression, and even mortality [66, 70]. In a previous report that analyzed in-hospital and 1-year post-discharge mortality, LDH (OR 1.012; 95% CI, 1.005–1.018, *p* < 0.001), platelet counts (OR 0.981; 95% CI, 0.967–0.995, *p* = 0.0100), and CRP levels (OR 0.985; 95% CI, 0.972–0.999, *p* = 0.030) were identified as relevant in the multivariate analysis for in-hospital mortality [51]. These were also included in the final multivariate model for late death, in spite of the different timeframes. LDH, a marker of cellular damage and hypoxia, has been considered a reliable indicator of pulmonary injury and a predictor of mortality in COVID-19 patients [71]. Elevated levels of this biomarker at admission have also been linked to prolonged hospitalization and ICU admission [72], and have been shown to predict severe disease based on the extent of lung lesions identified through chest CT scans [73]. CRP is a widely used marker for different kinds of infections, and high levels have been associated with inflammation, acute respiratory distress syndrome, and damage to organs such as the kidneys and the heart. Despite in some cases being considered an unspecific biomarker of inflammation, it has been extensively associated with COVID-19 severity and prognosis [74, 75]. SARS-CoV-2 infection is also associated with coagulopathy, often leading to thrombocytopenia, reflecting the broader impact of systemic inflammation. A reduction in platelet counts, particularly in the early stages of the disease, has been correlated with increased respiratory failure, greater disease severity, and a higher risk of mortality in COVID-19 patients [76].

## Limitations

The present study had some limitations that should be acknowledged. Firstly, the small sample size, particularly when even smaller samples were obtained for the study of mortality caused by COVID-19 and its timing, restricts the generalizability of these findings. The fact that all patients were admitted to two hospitals that belong to a single center is also a limitation, despite bias related to treatment and patient management being reduced. Hence, further research with larger and more diverse samples, for example, including data from centers across the entire country, is necessary to validate the findings obtained in this study. Nevertheless, our results contribute to a better understanding of the characteristics of the various COVID-19 waves in Portugal and the factors that influenced the observed trends. Additionally, they provide valuable insights for future research aimed at refining COVID-19-related prediction models by accounting for different variants and exploring the factors that affect early and late death in the ICU. Due to the limited research in this field, another limitation was the fact that all comparisons between the variables identified as significant in the models for early and late death with existing literature were conducted using data from different timeframes. In spite of the relevance of the mentioned comorbidities and biomarkers, it is important to highlight that most of the existing literature does not differentiate between the timeframes we selected for our study. Instead, the predictive power of such factors is typically assessed for the entire duration of the ICU admission. Moreover, the observational and retrospective nature of the study makes it more prone to missing data and unmeasured confounding factors that could have influenced the models’ results. For example, although having acquired data regarding patients’ medication, it was not possible to determine the timing of each administration, meaning this information was not accounted for in the logic regression models. This could potentially affect the interpretation of the effect of other variables in mortality. Due to the large quantity of medications and patient comorbidities, it also was not possible to differentiate specific treatments directed solely for the treatment of COVID-19 in each wave. Another limitation related to missing data was the inability to calculate or obtain disease severity scores, which could have provided invaluable information and helped account for additional confounding factors. For example, the Sequential Organ Failure Assessment (SOFA) score is commonly used to evaluate the extent of organ dysfunction and could have provided an objective measure of patients’ disease severity. The Acute Physiology and Chronic Health Evaluation (APACHE) score is another example, accounting for factors related to both acute disease (by including variables such as heart rate, blood pressure, and temperature) and chronic health conditions. Therefore, including these kinds of scores in future studies would be crucial to achieve more accurate predictions of COVID-19 patient outcomes. The same applies to data on secondary infections, which was also unavailable, as this information would have been invaluable in the multivariate analysis for understanding the relationship between certain blood biomarkers and the timing of death.

## Conclusion

Our results provide a good insight into the pandemic’s impact on the Portuguese population and the effectiveness of public health measures implemented through the different COVID-19 waves. We observed a significant decrease in the number of ICU admissions from the first to the sixth wave, with the second and third waves showing the highest mortality and risk of death. Furthermore, immunization may have contributed to a shift in the overall ICU scenario, by significantly reducing both patients’ median age and disease severity. Through multivariate logistic regression, we also identified that early and late COVID-19 mortality is influenced by different factors. Mortality within the first 72 h of ICU admission was primarily influenced by increasing age and levels of D-dimers and procalcitonin, whereas mortality after 72 h was associated with increasing age, hematologic cancer, increasing C-reactive protein and lactate dehydrogenase levels, and decreasing platelet counts. Overall, these findings emphasize the importance of further understating the factors that influence the timing of COVID-19-related mortality, while also considering the effect of different variants. Larger studies are needed to validate these novel results and better assess the risks associated with future variants.

## Supplementary Information

Below is the link to the electronic supplementary material.

Supplementary file1 (DOCX 216 KB)

## Data Availability

The datasets used for this study are available from the corresponding author on reasonable request.
