# Supplementary material for: Analysis of six consecutive waves of ICU-admitted COVID-19 patients: key findings and insights from a Portuguese population
Source: GeroScience. 2024 Nov 14;47(2):2399–422. doi: 10.1007/s11357-024-01410-x (PMC11979077; doi:10.1007/s11357-024-01410-x)

# Analysis of Six Consecutive Waves of ICU-Admitted COVID-19 Patients: Key Findings and Insights from a Portuguese Population

Cristiana P. Von Rekowski^1-3, *^, Iola Pinto^4,5^, Tiago A. H. Fonseca^1-3^, Rúben Araújo^1-3^, Cecília R.C. Calado^2,6^, Luís Bento^7,8^

^1^ NMS – NOVA Medical School, FCM – Faculdade de Ciências Médicas, Universidade NOVA de Lisboa, Campo Mártires da Pátria 130, 1169-056 Lisbon, Portugal.

^2^ ISEL – Instituto Superior de Engenharia de Lisboa, Instituto Politécnico de Lisboa, Rua Conselheiro Emídio Navarro 1, 1959-007 Lisbon, Portugal.

^3^ CHRC – Comprehensive Health Research Centre, Universidade NOVA de Lisboa, 1150-082 Lisbon, Portugal.

^4^ Department of Mathematics, ISEL – Instituto Superior de Engenharia de Lisboa, Instituto Politécnico de Lisboa, Rua Conselheiro Emídio Navarro 1, 1959-007 Lisboa, Portugal.

^5^ Center for Mathematics and Applications (NOVA Math), NOVA FCT – NOVA School of Science and Technology, Universidade NOVA de Lisboa, Largo da Torre, 2829-516 Caparica, Portugal.

^6^ iBB – Institute for Bioengineering and Biosciences, i4HB – The Associate Laboratory Institute for Health and Bioeconomy, IST – Instituto Superior Técnico, Universidade de Lisboa, Av. Rovisco Pais, 1049-001 Lisbon, Portugal.

^7^ Intensive Care Department, ULSSJ – Unidade Local de Saúde São José, Rua José António Serrano, 1150-199 Lisbon, Portugal.

^8^ Integrated Pathophysiological Mechanisms, CHRC – Comprehensive Health Research Centre, NMS – NOVA Medical School, FCM – Faculdade de Ciências Médicas, Universidade NOVA de Lisboa, Campo Mártires da Pátria 130, 1169-056 Lisbon, Portugal.

**Supplementary information**

1. **Tables**

Supplementary Table 1. Vaccines administered along the fourth, fifth, and sixth COVID-19 waves.

| **Waves**  Vaccines | Fourth (n=63) | Fifth (n=83) | Sixth (n=100)* | *p* value |
| --- | --- | --- | --- | --- |
| Pfizer | 33/63 (52.4) | 33/83 (39.8) | 53/100 (53.0) | 0.135 |
| AstraZeneca | 20/63 (31.7) | 30/83 (36.1) | 26/100 (26.0) |  |
| Moderna | 3/63 (4.8) | 9/83 (10.8) | 15/100 (15.0) |  |
| Janssen | 7/63 (11.1) | 11/83 (13.3) | 6/100 (6.0) |  |

*p* value obtained from Chi-squared test. *In the sixth wave, 101 patients received vaccines for the SARS-CoV-2, however there was no information about the kind of vaccine administered to one of the patients.

Supplementary Table 2. Median days between disease onset and ICU admission by ICU admission motive, and comparisons between COVID-19 waves.

| Admission Motive | Waves | Days between disease onset and ICU admission | *p* value |
| --- | --- | --- | --- |
| Infection by SARS-Cov-2 | **First (n=125)** | 8.0 (6.0-11.5) | <0.001* |
|  | **Second (n=176)** | 8.5 (5.0-11.0) |  |
|  | **Third (n=280)** | 8.5 (6.0-12.0) |  |
|  | **Fourth (n=147)** | 9.0 (7.0-12.0) |  |
|  | **Fifth (n=92)** | 6.0 (2.3-11.0) |  |
|  | **Sixth (n=50)** | 1.0 (0.0-7.75) |  |
| Other | **First (n=10)** | 7.5 (3.8-12.3) | <0.001** |
|  | **Second (n=10)** | 5.0 (0.0-9.5) |  |
|  | **Third (n=15)** | 2.0 (0.0-7.0) |  |
|  | **Fourth (n=15)** | 5.0 (0.0-9.0) |  |
|  | **Fifth (n=40)** | 1.0 (0.0-6.0) |  |
|  | **Sixth (n=81)** | 1.0 (0.0-1.0) |  |

*p* values were obtained from the Kruskal-Wallis test. * Pairwise comparisons tests revealed significant differences between wave 6 and all other waves, between wave 5 and waves 1, 3, and 4, and between wave 4 and wave 2 (all *p<*0.05). ** Pairwise comparisons tests revealed significant differences between wave 6 and all other waves, and between wave 5 and wave 1 (all *p<*0.05).

Supplementary Table 3. Correlation between the need for Invasive Mechanical Ventilation (IMV) and High-Flow Oxygen (HFO) in each of the six COVID-19 waves.

| **Waves**  Phi Coefficient | First | Second | Third | Fourth | Fifth | Sixth |
| --- | --- | --- | --- | --- | --- | --- |
| Value* | -0.681 | -0.872 | -0.806 | -0.749 | -0.463 | -0.199 |
| Significance | <0.001 | <0.001 | <0.001 | <0.001 | <0.001 | 0.023 |

*To assess the relationship between the two independent categorical/binary variables, in each wave, the phi coefficient was calculated. *p* values were considered significant at the 0.05 level (2-tailed). Strong correlations were considered for phi coefficients above 0.5.

Supplementary Table 4. Patients’ outcomes in the six COVID-19 waves, by age group.

**Age Group**

| Waves  **Variables** | Death in the ICU | ≥ 60 years | <60 years | *p* value |
| --- | --- | --- | --- | --- |
| First (n=136) | **Yes (n=29)** | 28 (96.6) | 1 (3.4) | <0.001 |
|  | **No (n=106)** | 64 (60.4) | 42 (39.6) |  |
| Second (n=185) | **Yes (n=66)** | 56 (84.8) | 10 (15.2) | <0.001 |
|  | **No (n=120)** | 66 (55.0) | 54 (45.0) |  |
| Third (n=294) | **Yes (n=106)** | 86 (81.1) | 20 (18.9) | <0.001 |
|  | **No (n=189)** | 73 (38.6) | 116 (61.4) |  |
| Fourth (n=162) | **Yes (n=26)** | 18 (69.2) | 8 (30.8) | <0.001 |
|  | **No (n=136)** | 36 (26.5) | 100 (73.5) |  |
| Fifth (n=133) | **Yes (n=34)** | 26 (76.5) | 8 (23.5) | 0.036 |
|  | **No (n=98)** | 55 (56.1) | 43 (43.9) |  |
| Sixth (n=131) | **Yes (n=22)** | 19 (86.4) | 3 (13.6) | 0.257 |
|  | **No (n=109)** | 82 (75.2) | 27 (24.8) |  |

*p* values obtained from Chi-squared or fisher’s exact tests, as appropriate.

Supplementary Table 5. Patients’ demographics, clinical and laboratorial data, considering their outcome and timing of death.

| Variables  **Group** | Discharged (n=758) | Deceased in the first 72h of ICU admission (n=39) | Deceased after the first 72h of ICU admission (n=224) |
| --- | --- | --- | --- |
| Age | 59.00 (47.00-70.00), n=758 (100.0%) | 70.00 (64.00-79.00), n=39 (100.0%) | 69.00 (63.00-76.00), n=224 (100.0%) |
| Sex (Male) | 519 (68.5%) | 27 (69.2%) | 161 (71.9%) |
| Origin (Portugal/Others) | 535 (70.6%) | 32 (82.1%) | 172 (76.8%) |
| Vaccine | 196 (25.9%) | 6 (15.4%) | 33 (14.7%) |
| Arterial Hypertension | 361 (47.6%) | 28 (71.8%) | 150 (67.0%) |
| Diabetes | 205 (27.0%) | 14 (35.9%) | 84 (37.5%) |
| Dyslipidemia | 157 (20.7%) | 11 (28.2%) | 67 (29.9%) |
| Obesity | 169 (22.3%) | 4 (10.3%) | 48 (21.4%) |
| Chronic respiratory disease | 79 (10.4%) | 4 (10.3%) | 41 (18.3%) |
| Stroke | 32 (4.2%) | 5 (12.8%) | 8 (3.6%) |
| Ischemic heart disease | 66 (8.7) | 5 (12.8%) | 26 (11.6%) |
| Congestive heart failure | 22 (2.9%) | 1 (2.6%) | 2 (0.9%) |
| Arrhythmias | 48 (6.3%) | 6 (15.4%) | 16 (7.1%) |
| Chronic kidney disease | 52 (6.9%) | 6 (15.4%) | 23 (10.3%) |
| Chronic liver disease | 17 (2.2%) | 1 (2.6%) | 6 (2.7%) |
| Solid Cancer | 42 (5.5%) | 1 (2.6%) | 21 (9.4%) |
| Hematologic cancer | 23 (3.0%) | 2 (5.1%) | 18 (8.0%) |
| Hypothyroidism | 32 (4.2%) | 1 (2.6%) | 11 (4.9%) |
| Autoimmune disease | 23 (3.0%) | 1 (2.6%) | 8 (3.6%) |
| HBP | 19 (2.5%) | 5 (12.8%) | 13 (5.8%) |
| Hyperuricemia | 28 (3.7%) | 1 (2.6%) | 8 (3.6%) |
| AIDS | 17 (2.2%) | 1 (2.6%) | 4 (1.8%) |
| History of organ transplant | 23 (3.0%) | 1 (2.6%) | 12 (5.4%) |
| Epilepsy | 15 (2.0%) | 1 (2.6%) | 3 (1.3%) |
| IMV | 456 (60.2%) | 33 (84.6%) | 208 (94.1%) |
| ECMO | 61 (8.0%) | 3 (7.7%) | 23 (10.4%) |
| HFO | 147 (19.4%) | 6 (15.4%) | 12 (5.4%) |
| pO2 (mmHg) | 101.00 (68.40-113.00), n=619 (81.7%) | 66.38 (49.30-111.50, n=25 (64.1%) | 85.75 (63.25-108.83), n=200 (89.3%) |
| Hemoglobin (x 10g/L) | 12.50 (10.93-13.60, n=668 (88.1%) | 11.00 (8.60-12.70), n=27 (69.2%) | 11.90 (10.20-13.30), n=204 (91.1%) |
| WBCs (x 10^9^/L) | 9.50 (6.73-13.44), n=668 (88.1%) | 10.41 (5.54-17.41), n=27 (69.2%) | 9.49 (6.84-13.74), n=207 (92.4%) |
| Lymphocyte counts (x 10^9^/L) | 0.75 (0.50-1.10), n=668 (88.1%) | 0.66 (0.42-1.26), n=27 (69.2%) | 0.57 (0.37-0.83), n=207 (92.4%) |
| LDH (U/L) | 446.00 (328.25-587.75), n=612 (80.7%) | 477.00 (347.00-579.00), n=27 (69.2%) | 535.00 (424.00-700.00), n=195 (87.1%) |
| Procalcitonin (ng/mL) | 0.18 (0.08-0.55), n=497 (65.6%) | 0.39 (0.08-15.80), n=21 (53.8%) | 0.35 (0.13-1.01), n=172 (76.8%) |
| CRP (mg/L) | 124.80 (55.10-208.30), n=671 (88.5%) | 116.80 (66.00-259.30), n=27 (69.2%) | 152.60 (102.30-233.00), n=207 (92.4%) |
| Platelet counts (x 10^9^/L) | 220.00 (171.50-293.00), n=665 (87.7%) | 169.00 (134.00-257.00), n=27 (69.2%) | 208.50 (152.00-277.50), n=206 (92.0%) |
| D-dimers (µg/L) | 517.50 (280.00-1308.75), n=508 (67.0%) | 981.00 (439.00-5945.00), n=25 (64.1%) | 777.00 (341.00-2224.00), n=187 (83.5%) |
| Creatinine (mg/dL) | 0.85 (0.71-1.22), n=669 (88.3%) | 1.85 (1.25-2.73), n=27 (69.2%) | 1.23 (0.83-1.83), n=206 (92.0%) |

Countinous variables are presented by their median and inter-quartile range (25th percentile; 75th percentile), along with the frequency and percentage of used cases, to account for missing values. Categorical variables are presented by their absolute frequencies and percentages (no missing values detected for these variables). Abbreviations: ICU – Intensive Care Unit; IMV – Invasive Mechanical Ventilation; ECMO – Extracorporeal Membrane Oxygenation; HFO – High Flow Oxygen; pO2 – Partial Pressure of Oxygen; WBC – White Blood Cell counts; LDH – Lactate Dehydrogenase; CRP – C-reactive Protein.

Supplementary Table 6. Univariate logistic regression results.

| Variables  **Group** | Discharged vs. Deceased in the first 72h of ICU admission | | | Discharged vs. Deceased after the first 72h of ICU admission | | |
| --- | --- | --- | --- | --- | --- | --- |
|  | Crude $\hat{OR}$ | 95%CI | *p value* | Crude $\hat{OR}$ | 95%CI | *p value* |
| Age | 1.070 | 1.041-1.100 | **<0.001** | 1.053 | 1.040-1.066 | **<0.001** |
| Categorized age | 6.909 | 2.673-17.854 | **<0.001** | 4.535 | 3.140-6.548 | **<0.001** |
| Sex | 0.965 | 0.481-1.938 | 0.921 | 0.850 | 0.611-1.181 | 0.332 |
| Wave Number (1) | Ref. | Ref. | 0.212 | Ref. | Ref. | **<0.001** |
| Wave Number (2) | 1.115 | 0.424-2.927 | 0.826 | 2.313 | 1.313-4.077 | **0.004** |
| Wave Number (3) | 0.711 | 0.273-1.857 | 0.487 | 2.510 | 1.478-4.263 | **<0.001** |
| Wave Number (4) | 0.197 | 0.041-0.945 | **0.042** | 0.742 | 0.383-1.440 | 0.378 |
| Wave Number (5) | 0.819 | 0.274-2.444 | 0.720 | 1.185 | 0.617-2.274 | 0.611 |
| Wave Number (6) | 0.368 | 0.095-1.425 | 0.148 | 0.509 | 0.234-1.108 | 0.089 |
| Vaccine | 0.521 | 0.215-1.263 | 0.149 | 0.495 | 0.331-0.742 | **<0.001** |
| IMV | 3.643 | 1.508-8.799 | **0.004** | 9.934 | 5.673-17.395 | **<0.001** |
| HFO | 0.756 | 0.311-1.837 | 0.537 | 0.235 | 0.128-0.432 | **<0.001** |
| Arterial Hypertension | 2.799 | 1.374-5.704 | **0.005** | 2.229 | 1.630-3.048 | **<0.001** |
| Diabetes | 1.511 | 0.770-2.963 | 0.230 | 1.619 | 1.182-2.217 | **0.003** |
| Dyslipidemia | 1.504 | 0.733-3.087 | 0.266 | 1.634 | 1.168-2.285 | **0.004** |
| Solid Cancer | 0.449 | 0.060-3.348 | 0.434 | 1.764 | 1.021-3.046 | **0.042** |
| Hematologic cancer | 1.727 | 0.392-7.605 | 0.470 | 2.792 | 1.478-5.274 | **0.002** |
| Chronic kidney disease | 2.469 | 0.989-6.159 | **0.053** | 1.554 | 0.928-2.601 | 0.094 |
| Chronic respiratory disease | 0.982 | 0.340-2.836 | 0.974 | 1.926 | 1.277-2.905 | **0.002** |
| pO2 (mmHg) | 0.984 | 0.971-0.997 | **0.020** | 0.999 | 0.995-1.002 | 0.422 |
| Hemoglobin (x 10g/L) | 0.759 | 0.641-0.899 | **0.001** | 0.915 | 0.852-0.983 | **0.016** |
| WBCs (x 10^9^/L) | 1.041 | 0.997-1.087 | 0.071 | 1.011 | 0.990-1.033 | 0.311 |
| Lymphocyte counts (x 10^9^/L) | 0.739 | 0.355-1.540 | 0.420 | 1.025 | 0.979-1.073 | 0.287 |
| LDH (U/L) | 1.000 | 0.999-1.001 | 0.857 | 1.001 | 1.000-1.001 | **0.002** |
| Procalcitonin (ng/mL) | 1.026 | 1.005-1.048 | **0.015** | 0.990 | 0.968-1.013 | 0.409 |
| CRP (mg/L) | 1.003 | 0.999-1.006 | 0.143 | 1.003 | 1.002-1.005 | **<0.001** |
| Platelet counts (x 10^9^/L) | 0.995 | 0.991-1.000 | **0.035** | 0.998 | 0.996-0.999 | **0.008** |
| D-dimers (mg/L) | 1.007 | 1.003-1.011 | **0.001** | 1.002 | 0.999-1.005 | 0.140 |
| Creatinine (mg/dL) | 1.249 | 1.080-1.444 | **0.003** | 1.189 | 1.079-1.311 | **<0.001** |

Solely variables with significant results in one of the two comparisons were displayed. For the logistic regression analysis, the reference category for the variable "Categorized age" was being age 60 or older, and for the variable "Sex," the reference category was female. Significant results are highlighted in bold. Abbreviations: ICU – Intensive Care Unit; $\hat{OR}$– Estimated Odds Ratio; IMV – Invasive Mechanical Ventilation; HFO – High Flow Oxygen; pO2 – Partial Pressure of Oxygen; WBC – White Blood Cell counts; LDH – Lactate Dehydrogenase; CRP – C-reactive Protein.

1. **Figures**

**Supplementary Figure 1.** Boxplots for patients’ number of days between disease onset and ICU admission, by wave, and significant results of multiple comparisons between them.


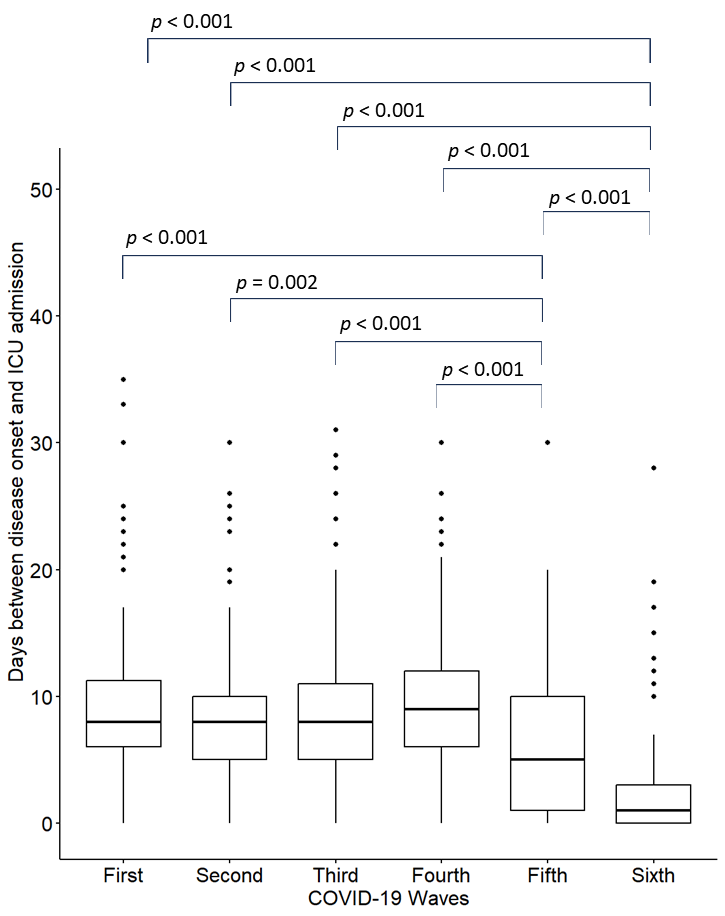


**Supplementary Figure 2.** Boxplots for patients’ ICU length of stay, by wave, and significant results of multiple comparisons between them.


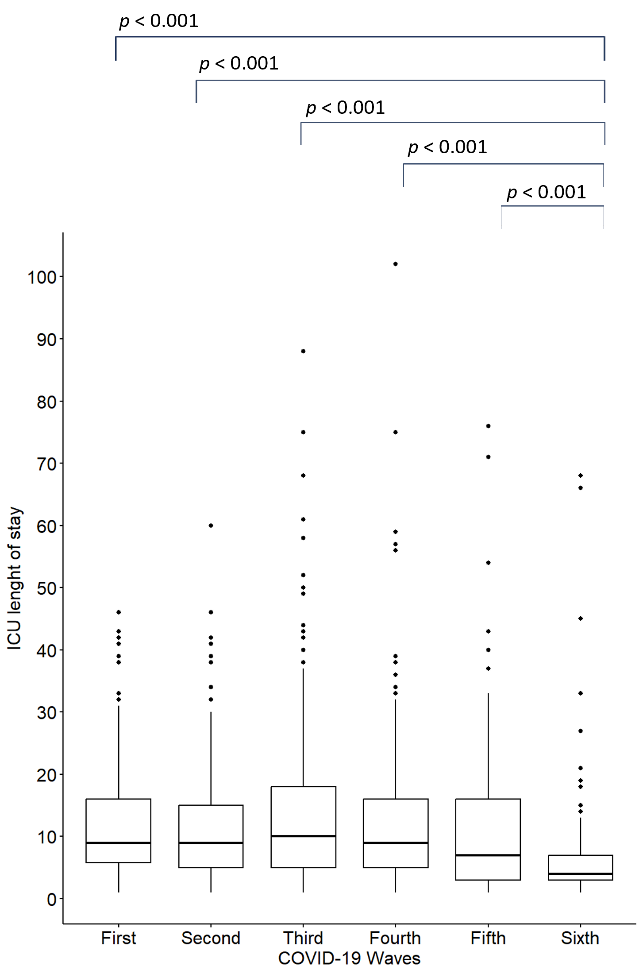

Supplement: Supplementary file 1 — Supplementary file1 (DOCX 216 KB) [file 11357_2024_1410_MOESM1_ESM.docx]
